# A genetic linkage map of black raspberry (*Rubus occidentalis*) and the mapping of *Ag*_*4*_ conferring resistance to the aphid *Amphorophora agathonica*

**DOI:** 10.1007/s00122-015-2541-x

**Published:** 2015-06-03

**Authors:** Jill M. Bushakra, Douglas W. Bryant, Michael Dossett, Kelly J. Vining, Robert VanBuren, Barbara S. Gilmore, Jungmin Lee, Todd C. Mockler, Chad E. Finn, Nahla V. Bassil

**Affiliations:** USDA-ARS National Clonal Germplasm Repository, 33447 Peoria Rd., Corvallis, OR 97333 USA; The Donald Danforth Plant Science Center, 975 N. Warson Rd., St. Louis, MO 63132 USA; B.C. Blueberry Council (in Partnership with Agriculture and Agri-Food Canada) - Pacific Agri-Food Research Centre, 6947 Hwy #7, P.O. Box 1000, Agassiz, BC V0M 1A0 Canada; Center for Genome Research and Biocomputing, Oregon State University, 3135 Agriculture and Life Science, Corvallis, OR 97331 USA; USDA-ARS National Forage Seed and Cereal Research Unit, 3450 SW Campus Way, Corvallis, OR 97331 USA; USDA-ARS Horticultural Crops Research Unit Worksite, 29603 U of I Ln., Parma, ID 83660 USA; USDA-ARS Horticultural Crops Research Unit, 3420 NW Orchard Ave., Corvallis, OR 97330 USA

## Abstract

*****Key message***:**

**We have constructed a densely populated, saturated genetic linkage map of black raspberry and successfully placed a locus for aphid resistance.**

**Abstract:**

Black raspberry (*Rubus occidentalis* L.) is a high-value crop in the Pacific Northwest of North America with an international marketplace. Few genetic resources are readily available and little improvement has been achieved through breeding efforts to address production challenges involved in growing this crop. Contributing to its lack of improvement is low genetic diversity in elite cultivars and an untapped reservoir of genetic diversity from wild germplasm. In the Pacific Northwest, where most production is centered, the current standard commercial cultivar is highly susceptible to the aphid *Amphorophora agathonica* Hottes, which is a vector for the *Raspberry mosaic virus* complex. Infection with the virus complex leads to a rapid decline in plant health resulting in field replacement after only 3–4 growing seasons. Sources of aphid resistance have been identified in wild germplasm and are used to develop mapping populations to study the inheritance of these valuable traits. We have constructed a genetic linkage map using single-nucleotide polymorphism and transferable (primarily simple sequence repeat) markers for F_1_ population ORUS 4305 consisting of 115 progeny that segregate for aphid resistance. Our linkage map of seven linkage groups representing the seven haploid chromosomes of black raspberry consists of 274 markers on the maternal map and 292 markers on the paternal map including a morphological locus for aphid resistance. This is the first linkage map of black raspberry and will aid in developing markers for marker-assisted breeding, comparative mapping with other *Rubus* species, and enhancing the black raspberry genome assembly.

**Electronic supplementary material:**

The online version of this article (doi:10.1007/s00122-015-2541-x) contains supplementary material, which is available to authorized users.

## Introduction

Genetic linkage map construction of rosaceous crops has been used to understand genetics and as a precursor to enabling molecular breeding for about 20 years. The earliest maps made during the 1990s were constructed mainly by using isozymes, random amplification of polymorphic DNA (RAPD), restriction fragment length polymorphism (RFLP), and morphological markers (Chaparro et al. 1994; Foolad et al. 1995; Hemmat et al. 1994; Rajapakse et al. 1995; Stockinger et al. 1996; Viruel et al. 1995). Advancements in DNA technology in the 2000s led to the rapid development of simple sequence repeat (SSR) markers for de novo map construction (Castro et al. 2013; Celton et al. 2009; Dirlewanger et al. 2004; Fernández-Fernández et al. 2008; Gisbert et al. 2009; Graham et al. 2004; Hibrand-Saint Oyant et al. 2008; Olmstead et al. 2008) as well as their incorporation into existing maps (Aranzana et al. 2003; Dirlewanger et al. 2006; Etienne et al. 2002; Liebhard et al. 2003; Paterson et al. 2013; Pierantoni et al. 2004; Silfverberg-Dilworth et al. 2006; Stafne et al. 2005; Vilanova et al. 2008; Woodhead et al. 2008, 2010). Additional technological advances in high-throughput detection of single-nucleotide polymorphic (SNP) loci using arrays, or genotyping by sequencing (GBS), and the associated improvement of data analysis have made SNP markers increasingly useful for genetic map construction. Recently, linkage maps for several members of the Rosaceae have been constructed using SNP array technology (Antanaviciute et al. 2012; Clark et al. 2014; Frett et al. 2014; Klagges et al. 2013; Montanari et al. 2013; Pirona et al. 2013; Yang et al. 2013).

The genus *Rubus* L. (Rosaceae, *Rosoideae*) has an estimated 750 species distributed world-wide (Alice and Campbell 1999; Thompson 1995). Of these, three are of particular commercial importance, red raspberry (*R. idaeus* L., subgenus *Idaeobatus* Focke), blackberry (*Rubus* sp., subgenus *Rubus* L.), and black raspberry (subgenus *Idaeobatus*). Genetic linkage maps have been constructed for tetraploid blackberry (Castro et al. 2013), diploid red raspberry (Sargent et al. 2007; Ward et al. 2013; Woodhead et al. 2010), and an interspecific cross between diploid red raspberry and diploid black raspberry (Bushakra et al. 2012). While blackberry and red raspberry are highly heterozygous, black raspberry, particularly *R. occidentalis*, is highly homozygous (Dossett et al. 2012b). Genetic improvement of blackberry and red raspberry through breeding has been a continual process for decades. For example, from 1994 to 2014, the American Pomological Society’s Fruit and Nut Variety Registry Lists 38–47 (Clark and Finn 1999, 2002, 2006; Clark et al. 2008, 2012; Daubeny 1997a, b, 1999, 2000, 2002, 2004, 2006, 2008; Finn and Clark 2000, 2004, 2014; Finn et al. 2010; Moore and Kempler 2010, 2012, 2014) records the release of 75 blackberry and hybrid berry and 189 red raspberry cultivars and only three black raspberry cultivars (‘Pequot’, ‘Niwot’, and ‘Explorer’). In addition, ‘Earlysweet,’ a selection derived from a purported cross between *R. occidentalis* and the western black raspberry, *R. leucodermis* Dougl. ex Torr. & Gray, was released in 1998 (Galletta et al. 1998). Black raspberry figures prominently in the pedigrees of many of the red raspberry cultivars released between 1994 and 2014. Difficulties in improving black raspberry through breeding were first reported by Slate (1933) while attempting to improve purple raspberries. Crossing with other species was proposed as a way to increase genetic diversity in cultivated black raspberry (Drain 1956; Hellman et al. 1982; Slate and Klein 1952), but has met with limited success. Low genetic diversity was proposed by Ourecky (1975) as the main reason for lack of development of improved cultivars.

More recent interest in improving black raspberry has been driven by research and commercial interest into its bioactive compounds and their influence on human health, specifically modulation of cancer cell proliferation, inflammation, cellular death, oxidation, etc. (Stoner et al. 2007). Since the 1940s, Oregon has been the primary commercial production region of black raspberry in North America. In 2014, 1650 acres were harvested that earned growers a utilized production value of over US$16.8 million (Anonymous 2015). One hindrance to expanding production is susceptibility of the predominant cultivar Munger to the *Raspberry mosaic virus* complex vectored by the North American large raspberry aphid, *Amphorophora agathonica* Hottes (Dossett and Finn 2010). Infection causes rapid decline of plantings, often with field replacement necessary after only three or four growing seasons (Halgren et al. 2007). In contrast, under perennial production in open fields for processed fruit, plantings of current cultivars of red raspberry are typically kept in the field for 7–8 growing seasons, and plantings of blackberry cultivars can last many decades (C.E. Finn, personal communication). Selection for cultivars with resistance to *A. agathonica* could significantly increase the longevity of the plants, reduce insecticide use, and therefore improve profitability for the grower and quality of the environment.

A low level of genetic diversity in cultivated black raspberry has been found using molecular tools. Weber (2003), using RAPD markers in 16 black raspberry cultivars, determined a level of similarity of 81 %. Two wild accessions and five elite genotypes accounted for more than 50 % of the similarity, while the remaining 11 cultivars shared 92 % similarity compared to 70 % similarity among red raspberry cultivars found by Graham et al. (1994). In 2005, Lewers and Weber used SSR markers from red raspberry and strawberry to evaluate an F_2_ population of a red raspberry × black raspberry cross and found that the homozygosity of the black raspberry clone used was 80 % and only 40 % in the red raspberry clone used. However, wild populations of black raspberry show greater genetic diversity. For example, Nybom and Schaal (1990) sampled black raspberry plants along a roadside in Missouri that were then analyzed by RFLP. They found 17 informative fragments that identified 15 genotypes in the 22 samples collected. Dossett et al. (2012b) used SSR markers to examine the genetic diversity among cultivars and wild germplasm. They found that the diversity at 21 loci was much higher among wild germplasm than in the elite cultivars, and that six elite cultivars were identical at these 21 loci.

Genetic diversity in wild black raspberry germplasm as detected by molecular tools (Dossett et al. 2012b; Nybom and Schaal 1990) and through breeding experiments (Dossett et al. 2008) is currently untapped. To address this, Dossett and Finn (2010) canvassed the native range of *R. occidentalis* collecting seed, which was subsequently germinated and evaluated for multiple traits including aphid resistance. From this study, three of 132 wild populations were determined to segregate for resistance to *A. agathonica*. Two populations, ORUS 3817 collected from Maine, and ORUS 3778 collected from Ontario, Canada, were subsequently used to develop populations for genetic mapping and phenotypic analysis. F_1_ progeny of susceptible cultivars Munger and Jewel crossed with individuals from ORUS 3778 and ORUS 3817 were all resistant to aphids under greenhouse conditions suggesting that the alleles for resistance are dominant and that ORUS 3778 (*Ag*_*4*_) and ORUS 3817 (*Ag*_*5*_) are homozygous for their respective alleles. Dossett and Finn (2010) originally identified one of the susceptible cultivars used in the crosses as ‘Black Hawk’, however, subsequent fingerprinting work found it to be ‘Jewel’ (Dossett et al. 2012a).

In this paper, we report the analysis of population ORUS 4305, an F_1_ black raspberry population, raised as one of several populations to investigate genetic sources of resistance to the aphid *A.* *agathonica* with the intent of mapping the aphid resistance allele *Ag*_*4*_. To quickly and efficiently generate markers for mapping we have employed GBS following the protocol established by Elshire et al. (2011) with modifications for *Rubus* (Ward et al. 2013), and anchored the map with SSR markers from a variety of sources. We have placed the phenotypic character of aphid resistance on this linkage map which covers the seven *Rubus* linkage groups (RLG) as defined by Bushakra et al. (2012).

## Methods

### Plant material

**Fig. 1:**
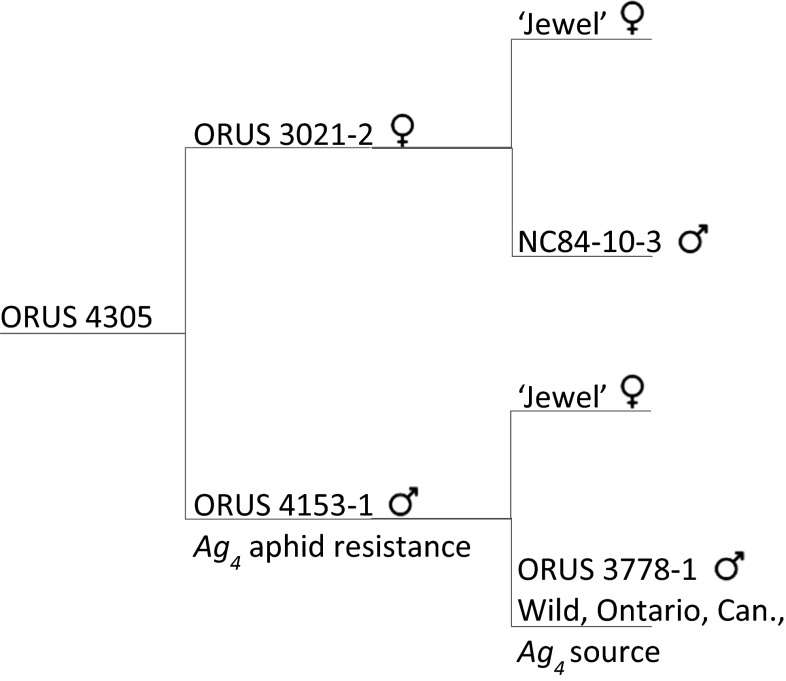
Pedigree of mapping population ORUS 4305. Population ORUS 4305 is derived from a wild-collected accession from Ontario, Canada (ORUS 3778-1), that exhibited resistance to the North American large raspberry aphid that was crossed with aphid-susceptible ‘Jewel.’ One of the progeny from that cross, ORUS 4153-1 with proposed genotype *Ag*_*4*_ *ag*_*4*_ representing the proposed gene conferring resistance, was used as the male parent and crossed with aphid-susceptible ORUS 3021-2

A full-sib (F_1_) family of 115 individuals was developed from the controlled cross of ORUS 3021-2 (female, susceptible to aphids, postulated genotype *ag*_*4*_*ag*_*4*_) × ORUS 4153-1 (male, resistant to aphids, postulated genotype *Ag*_*4*_*ag*_*4*_; Fig. 1). The source of this resistance is from ORUS 3778-1, an accession from wild seed collected in Ontario, Canada (Dossett and Finn 2010). Progeny from this cross were screened for aphid resistance as small seedlings in the greenhouse as described by Dossett and Finn (2010) and followed the expected 1:1 segregation ratio (56 resistant, 59 susceptible, *χ*^2^ = 0.08, *P* = 0.78).

### DNA extraction

Leaf samples were collected, bagged, kept cool, and transported to the laboratory. Leaf tissue aliquots of 30–50 mg were placed in a cluster tube (Corning Life Sciences, Tewksbury, MA, USA) containing a 4-mm stainless steel bead (McGuire Bearing Company, Salem, OR, USA). The samples were frozen in liquid nitrogen and stored at −80 °C until extraction. Frozen tissue was homogenized using the Retsch^®^ MM301 Mixer Mill, (Retsch Inc., Hann, Germany) at a frequency of 30 cycles/s using three 30 s bursts. The E-Z 96^®^ Plant DNA extraction kit (Omega Bio-Tek, Norcross, GA, USA) was used as previously described (Gilmore et al. 2011).

### DNA quantification and quality

Genomic DNA was quantified using Quant-iT™ Picogreen^®^ dsDNA Assay kit (Invitrogen, Eugene, OR, USA) following manufacturer’s instructions modified to 100 μl and compared against a λ standard DNA dilution series with a Victor^3^V 1420 Multilabel Counter (PerkinElmer, Downers Grove, IL, USA), followed by visualization on 1 % agarose gel in 1× TBE (Tris/Borate/EDTA) stained with ethidium bromide. Samples were stored at −20 °C prior to use.

### Marker sources

SSR primer pairs were selected from multiple sources (Table 1). Markers derived from GBS were coded as S with a number indicating the scaffold followed by an underscore and a number indicating the physical SNP position on the scaffold (i.e., S75_381030) (Bryant et al. 2014). Markers developed from the sequencing of paired-end short reads were coded with Ro (*R. occidentalis*) or Ri (*R. idaeus*) followed immediately by a number (i.e., Ro11481, Ri13528) (Dossett et al. in press). All other markers are from published sources as indicated in Table 1. Ag4_AphidR is a phenotypic marker for aphid resistance.

**Table 1:** Transferable locus primer sequences used to construct the genetic linkage maps for black raspberry F_1_ population ORUS 4305

Locus	RLG	3021-2 allele sizes	4153-1 allele sizes	Repeat motif	Forward primer sequence	Reverse primer sequence	Source
*ERubLR_SQ07*-*3_C07_HRM_RLG7*	*7*	NA	NA	NA	GGATCAAGGAGTGAGGATGG	CCGTGGTGGTTGTTATGTTG	Bushakra et al. (2012)
*Ri_1B16_HRM_RLG7*	*7*	NA	NA	NA	CTTGGGCAGCTTTAGCCTTT	AAGAAGAAGGGTGGGTTTCA	
*Ru_EE284382_HRM_RLG5*	*5*	NA	NA	NA	ACGGAGGATGACAGAGAACC	AGGTGAGGTGGGAGATGATG	
Ro_CBEa0001L24_SSR	2	275, 277	277, 279	(CCA)_4_–(TA)_7_	M13-TAAAGAAAGGGGTTGTTGCG	GACGTCTCCATTGGGAAGAA	Unpublished
Ro_CBEa0002P01b_SSR	1	226, 228	228	(TC)_4_–(CT)_7_	M13-CCCTCCCTCTCTCCAGTTTC	GCGCTTGAGCATCAAATGTA	
Ro_CBEa0003K17_SSR	1	307	307, 309	(TC)_6_	M13-GGAAAGAAAACCCTAGCCGA	CTTACGCTTCTTGGCTCCAC	
Ro_CBEa0004G23_SSR	4	509	507, 509	(AG)_8_	M13-GACGCGGTGAGATTTTGATT	GTTCCCTTTGCTTTGAGACG	
Ro_CBEa0009J05_SSR	4	292	292, 294	(CA)_4_–(GA)_3_	M13-CCAAGTCCAACCACTCACAC	TTTGCTCGTCGTACTCATCG	
*RhM003_SSR_RLG3*	*3*	227, 236	236, 238	(TG)_10_	M13-CCATCTCCAATTCAGTTCTTCC	AGCAGAATCGGTTCTTACAAGC	Castillo et al. (2010)
RiM017_SSR	4	212, 214	212	(TG)_6_	M13-GAAACAGGTGGAAAGAAACCTG	CATTGTGCTTATGATGGTTTCG	
Rh_MEa0006bG05_SSR	6	294, 303	294	(AAG)_8_	M13-GAAGCAGCAGCAAGACCTTT	GTTCAGGCCAGTCAATGTCA	Castro et al. (2013)
Rub1C6_SSR	6	244, 260	262, 268	(CT)_15_	**D4-TCTGCCTCTGCATTTTACACAG**	GTTTAGGTAAGCAATGGGAAAGCTC	Dossett and Finn (2010)
Ro_CBEa0010N20_SSR	4	114, 118	118	(GA)_9_	M13-GGGGGCTTTACATCATCATT	TTCGTAGTCTTGCCCTTGCT	Dossett et al. (2012b)
Ro_CBEa0011M11_SSR	5	243, 245	241, 243	(AG)_14_	M13-GGGCATGAACCACATAAAGG	TCCATTTCCAAAACACATTGA	
Ri10139_SSR	7	295	295, 314	(TC)_8_	M13-GTCTCGGCCGAATAATAAACAA	CACGAAGAACAACGAGAGAAAA	Dossett et al., in press
Ri11086_SSR	2	268, 300	268, 302	(TC)_9_	M13-AAAATTCTGATTGGGCCGAC	ACAACACGAAGAACACGAGAGA	
Ri11795_SSR	3	299, 313	299, 313	(GAA)_8_	M13-ATCCAACCCTTCATTCTCTGTT	GCGAAGACGAGGAAGATGAAT	
Ri12319_SSR	3	304	292, 304	(TCA)_7_	M13-GAGTCTGATATCAAGCAGCCCT	AAAGGTAGAAGTGGAGGACTCA	
Ri13528_SSR	7	460, 463	463	(TC)_10_	M13-CTCTGCTACAACCCAACGAGTC	GGCAATTTGGAGATTTCTTGCT	
Ri14075_SSR	2	351, 355	357, 359	(TC)_8_	M13-ACAAATTCCAGTCAGTCCATGC	CCAGACGCATTAAATCTGTCAC	
Ri16959_SSR	7	283, 286	286	(TC)_6_	M13-AAAATGTGATTGAGCCGACG	GGGAAAACTGAAGAACACGAAG	
Ri18886_SSR	1	308, 317	314, 317	(ATG)_9_	M13-CCCAAAGGACAGAAGTATGGAC	CGGGTCTTACAGGCAAGTGATA	
Ri20047_SSR	5	376, 393	376, 405	(CT)_9_	M13-CCCTGTTTGATCTATTCAATCCC	GAGGAGCAGCTTGTCGAGAT	
Ri20466_SSR	2	369	369, 374	(GA)_10_	M13-GGTTTTCTGGGAAAAACAGAAG	CGCGTTTTCACTGTTCACTTTA	
Ri3758_SSR	2	392, 406	400, 406	(AT)_7_	M13-GTTTTGCCTACGGACTTGAATC	TCTATCTCTCCGTTGTGGATTT	
Ri5037_SSR	2	336, 339	336, 339	(GAA)_6_	M13-CACGAGTAACACTCCCAAATGA	TCTTGGAATTGGGGTTATTCTG	
Ro10488_SSR	2	119	121, 131	(TC)_9_–(TC)_14_	M13-AGGGTGCGTGTCAGAAGTAAGT	GCTGATAGTGGGGTTTGGATAA	
Ro1079_SSR	4	223, 225	223	(TA)_6_	M13-AAAATGGAGACTAGATCCAGCG	GGCAGAGATTTGAGGTTTCTGA	
Ro11481_SSR	6	156	156, 158	(AG)_6_–(TA)_6_–(AT)_5_	M13-AAGATAAGAGGAGAAGTGCGGA	CTGTTTCCAGCAAACCTAACCT	
Ro12112_SSR	6	157, 167	140	(TC)_8_	M13-TACTCCCAAAAACCCAGAATTG	GTCTGAGCAGAAATGGGAAATC	
Ro14925_SSR	7	117	114, 117	(TGT)_7_	M13-AGCTGGTCGAAGAAGGTTTATG	AACTTTCTCCCGTTCTCCTAGC	
Ro15590_SSR	6	179, 211	201, 205	(GA)_6_	M13-GGAGCAAGAAGCCTTGAAGATA	GTTGCCTCTGGATTGCTTTTAT	
Ro16697_SSR	4	152, 160	160	(AT)_8_–(TA)_6_	M13-CCAGTGAGTGAGCCTTGAGATA	ATTTGGAAGGAATACGGAACCT	
Ro1682_SSR	3	119, 125	121	(AT)_6_	M13-AGGAGCGATGTTATAGGCATGT	TAGAGGGAGAAAAAGGGAGTGC	
Ro17045_SSR	3	164	164, 167	(TGA)_8_	M13-TCCAACATTGGTGACAGTTTTC	ACTTTTGCATCTGCTTCATCTG	
Ro17803_SSR	2	139, 147	139, 141	(TA)_10_	M13-GCCCGATAGATTAAAAGGGAAA	GTTCAGAATGCAGTTGAAACCA	
Ro18036_SSR	1	104, 119	119	(CCT)_8_	M13-CTTCTTGGGACGAAAAACAAAC	CTGTGGATTCAGACGAAGATGA	
Ro19042_SSR	6	201	196, 201	(GA)_6_	M13-GGGTATATTCCAAAACCCCAAT	TGGGTTTCAAAGGTCAATCTCT	
Ro20267_SSR	4	159	153, 159	(TGA)_8_	M13-GAACCAAAGCTTTTGATTGGTC	GTTGGATTTCATGGAAAGTGTC	
Ro2173_SSR	4	199, 240	203, 221	(TTA)_8_	M13-TATTGGGAGTGAAAGAGCCCTA	GGTGTATTTTAATGCGGTCACA	
Ro2432_SSR	5	114, 116	114	(TC)_8_	M13-CGGATGAATTTAAGAAAGCTGG	CTTCTCAAGAACACGGCGAT	
Ro2579_SSR	4	181	179, 181	(CA)_10_	M13-TTTTATATGCTTGTCCCACACG	ATTATAGAAATTGGGCGCACTC	
Ro2827_SSR	5	133, 141	137, 141	(CT)_6_	M13-GCGTCTGCTTTCTTCTCAGTCT	GAGCGCAGAAGCAGACTTATCT	
Ro3003_SSR	5	145, 199	145, 152	(GA)_6_–(GA)_7_	M13-ACGTTGATCATAGCCTCCAAAT	CTTCCCATAGCAACTCTATCCC	
Ro3017_SSR	3	159, 173	161	(GA)_7_	M13-CAACCGCTTTAATGAAGTGTGA	GCACAAGTAGCACAACTCAACA	
Ro3237_SSR	1	131, 135	133, 135	(TA)_7_	M13-AACCCAAAGCTTTCCTTCTTGT	ATTGGCAGGCTTTCCTTACATA	
Ro3981_SSR	6	115, 117	115	(TG)_7_–(TG)_7_	M13-GATCTCTGATTCCCGCATTATT	AAATGTCCTTCCTGATGATTGG	
Ro4104_SSR	7	181, 185	181, 183	(TA)_7_	M13-AAAGCTTCCTCATTTTGTGAGC	ATGATATGACGGCTGAGATCAA	
Ro4261_SSR	4	204, 219	204, 219	(TTC)_9_	M13-AATAGCATGGAATCCACTCACC	TCTCATTCCAGATGGGTTATCA	
Ro4345_SSR	5	108, 114	114	(TC)_7_	M13-TTACAGCAATTGAAGGATGAGC	AAAGAAATAGGGAAAGGGGGAG	
Ro4532_SSR	6	210, 213	210, 213	(TTG)_6_	M13-AGTTCATCAATTTGAGGGATGG	TCGATGATCATATCATTCCACC	
Ro5263_SSR	6	201, 203	201	(GA)_6_	M13-AACCTTTTGCGTTTGATACTCC	TTTGTTTGCCTTAGAGTCCTCC	
Ro5378_SSR	4	207	191, 207	(TA)_8_	M13-TCTTCACACATGTCCACTGGTT	TCAGCTGAGTTTTTGCAGAGAT	
Ro6594_SSR	1	171, 177	171	(TTC)_9_	M13-TTTGAGAGGACGAATGTCGTTA	CTGTAATACTAGGCTCCACCGC	
Ro7270_SSR	3	168, 171	177	(GAA)_8_	M13-CTCAGGAAACCGTCATACTTCC	TGGTCTTCCATAACCCTTCAGT	
Ro8167_SSR	6	94, 96	96	(TC)_6_	M13-CAATTGCACATAACCCATCATC	GAAGGAATGCAAAACCAGAAAG	
Ro8486_SSR	2	172, 178	172	(CT)_9_	M13-TCGCGCTGATAGTGTTTCATAC	AAGGAATGAAATAGGGACGGTT	
Ro9206_SSR	5	135, 139	126, 137	(AT)_8_	M13-ACAGTTCCTACAAAGGATCGGA	CAAGATTGTCACGTACTCGGAA	
Ro9324_SSR	1	156, 164	164, 201	(AG)_7_	M13-CCTACTTTCAAAGCCCATTTTG	GCAATCACACATTAAAAGGTCC	
Ro942_SSR	1	155, 161	161, 181	(GAA)_7_	M13-AATCGTCGCCTGCAATATTTAC	CAAATTCGACACCACCTATCAG	
*Rubus110a_SSR_RLG4*	*4*	187, 207	203, 205	(TC)_8_	M13-AAACAAAGGATAAAGTGGGAAGG	TGTCAGTTGGAGGGAGAACA	Graham et al. (2004)
*Rubus116a_SSR_RLG4*	*4*	222, 224	218, 224	(CT)_12_–(T)_10_	M13-CCAACCCAAAAACCTTCAAC	GTTGTGGCATGGCCTTTTAT	
*Rubus126b_SSR_RLG2*	*2*	171	157, 171	(CT)_31_–(CA)_22_	M13-CCTGCATTTTTCTGTATTTTGG	TCAGTTTTCTTCCCACGGTTA	
*Rubus16a_SSR_RLG6*	*6*	170, 172	164, 166	(AT)_8_(GT)_11_	M13-TGTTGTACGTGTTGGGCTTT	GGGTGTTTGCCAGTTTCAGT	
Rubus223a_SSR	6	158, 162	158	(AT_)4_–(TA)_8_–(AT)_10_	M13-TCTCTTGCATGTTGAGATTCTATT	TTAAGGCGTCGTGGATAAGG	
Rubus26a_SSR	4	139, 141	137, 143	(CT)_11_–(CA)_29_	M13-AACACCGGCTTCTAAGGTCT	GATCCTGGAAAGCGATGAAA	
*Rubus270a_SSR_RLG3*	*3*	182, 184	184, 186	(GA)_10_	**D3-GCATCAGCCATTGAATTTCC**	CCCACCTCCATTACCAACTC	
*Rubus275a_SSR_RLG5*	*5*	139, 165	165	(AG)_27_	M13-CACAACCAGTCCCGAGAAAT	CATTTCATCCAAATGCAACC	
Rh_MEa0002cA01_SSR	2	268, 274	268, 272	(CT)_17_	M13-CCCCAAACTCCAAAATCTCA	TTCTGCTCATCTTTGGGGTC	Lewers et al. (2008)
*Rh_MEa0007aB01_SSR_RLG5*	*5*	148, 154	148, 152	(CT)_15_	M13-TGGTGGTTCACCGTTCACTA	GAAATGCTTGAAGCCGAGAG	
Rh_MEa0013bG01_SSR	2	248, 250	248	(GA)_38_	M13-CCCTCCATCTCCACCATAAA	GTAAGGCCACCCCATTGAG	
Rh_MEa0013cF08_SSR	1	254, 256	254, 266	(TC)_15_	M13-TTTGTCTCCGTCTTTTTGCC	CCTCCGAAGAAAAACAGCAG	
*ERubLRSQ_07*-*4_D05_SSR_RLG6*	*6*	260	260, 266	(AGC)_7_	M13-CTTCTTTCCAACCGATTTC	ACGAATTGATTTCATCAACC	Woodhead et al. (2008)

Locus names prefaced with Ro were derived from black raspberry (*Rubus occidentalis* L.), those prefaced with Ri and Rubus were derived from red raspberry (*R. idaeus* L.), those prefaced with Rh were derived from blackberry (*Rubus* sp.). Names in italics designate those markers that were used to anchor the linkage groups and were selected because they map in multiple *Rubus* linkage maps. Two markers (Rub1C6 and Rubus270a) were designed with a fluorescent tag on their forward primer. Each entry includes the linkage group to which the locus mapped, the allele size in population ORUS 4305, the repeat motif, the forward and reverse primer sequence, and source. The sequence of the M13 tag is 5′-TGTAAAACGACGGCCAGTAGC

An additional 26 SSR and two high-resolution melting (HRM) markers that mapped in multiple populations were identified from the literature (Bushakra et al. 2012; Castillo et al. 2010; Castro et al. 2013; Fernández-Fernández et al. 2013; Graham et al. 2004; Sargent et al. 2007) with the intention of anchoring and orienting the linkage groups to published maps (Table 2).

**Table 2:** Summary of loci mapped in F_1_ population ORUS 4305

**Genotyping by sequencing (GBS)**	
Total number of GBS SNP identified over three sequencing runs	7911
Number of monomorphic or ambiguous loci	3472
Number of loci heterozygous in both parents	921
Number of loci heterozygous in ORUS 3021-2	318
Number of loci heterozygous in ORUS 4153-1	326
Total scaffolds represented	356
Scaffolds mapping to multiple RLG	13
Total number of GBS SNP mapped	399
**Simple sequence repeat (SSR)**	
Total number of loci screened	552
Number of monomorphic or ambiguous loci	235
Number of loci that failed	118
Number of loci that are heterozygous in both parents	138
Number of loci heterozygous in ORUS 3021-2	29
Number of loci heterozygous in ORUS 4153-1	32
Number of loci mapped	70
**High-resolution melting (HRM)**	
Total number of loci screened	80
Number of monomorphic or ambiguous loci	69
Number of loci that failed	0
Number of loci heterozygous in ORUS 3021-2	7
Number of loci heterozygous in ORUS 4153-1	4
Number of loci mapped	3
Anchor loci (26 SSR + 2 HRM)	28
Not mapped	16
Mapped	12

Genotyping by sequencing (GBS) single nucleotide polymorphic (SNP) loci were generated by DNA digestion and subsequent high-throughput sequencing. Data were analyzed for mapping using the TASSEL computer software provided through the Buckler Lab for Maize Genetics and Diversity. Simple sequence repeat (SSR) and high-resolution melting (HRM) loci were derived from a number of sources

### DNA amplification of SSR markers

DNA amplification was performed on a PTC-225 gradient thermal cycler (Bio-Rad, Hercules, CA, USA), a Dyad Peltier thermal cycler (Bio-Rad, Hercules, CA, USA), an Eppendorf Mastercycler (Eppendorf, Hamburg, Germany), or a Nexus (Eppendorf, Hamburg, Germany). A fluorescent labeling polymerase chain reaction (PCR) protocol (Schuelke 2000) was used for most SSR primer pairs. The forward (F) primer of each pair was extended on the 5′-end with an M13-TGTAAAACGACGGCCAGTAGC sequence tag to which a universal M13-tagged fluorescent dye label (WellRed D2, D3, D4; Integrated DNA Technologies, Inc., Coralville, IA, USA) annealed. The touch-down PCR protocol began with an initial denaturation for 3 min at 94 °C followed by 10 cycles of 94 °C for 40 s, 65 °C (decreasing 1 °C every cycle) for 45 s, 72 °C for 45 s; 20 cycles of 94 °C for 40 s, 52 °C for 45 s, 72 °C for 45 s; 10 cycles of 94 °C for 40 s, 53 °C for 45 s, 72 °C for 45 s; followed by a final extension of 72 °C for 30 min. Reactions were performed in a final volume of 15 μl consisting of 6 ng template DNA, 1× PCR buffer, 2 mM MgCl_2_, 200 μM dNTP, 0.5 μM reverse primer, 0.12 μM M13-tagged forward primer, 0.5 μM WellRed labeled M13 primer (D2, D3 or D4), and 0.025 U GoTaq^®^ Hot Start Polymerase (Promega Corporation, Madison, WI, USA). For a few SSR primer pairs, the 5′-end of the F primer was fluorescently labeled (WellRed D2, D3, or D4). The PCR protocol used for labeled F-primers began with an initial denaturation for 3 min at 94 °C followed by 35 cycles of 94 °C for 40 s, appropriate annealing temperature for 40 s, 72 °C for 30 s; followed by a final extension of 72 °C for 30 min. The reverse primer for Rub1C6 was PIG-tailed with 5′-GTTT-3′ (Brownstein et al. 1996) to minimize the occurrence of split peaks and difficulties encountered in automated fragment analysis following capillary electrophoresis.

### Capillary electrophoresis of SSR markers

Success of the PCR was confirmed by 2 % agarose gel electrophoresis. Up to six fragments were pooled based on dye and predicted fragment size and separated on a Beckman CEQ 8000 capillary genetic analyzer (Beckman Coulter, Fullerton, CA, USA). Separation was followed by analysis of allele size and marker visualization using the fragment analysis module of the CEQ 8000 software.

### High-resolution melting

The HRM technique (Wittwer et al. 2003) was used to amplify markers from Bushakra et al. (2012). Reactions were performed on PTC-225 gradient thermal cycler (Bio-Rad, Hercules, CA, USA), followed by HRM on the LightScanner^®^ System (BioFire Defense, Salt Lake City, UT, USA). Reactions were performed in a final volume of 10 μl consisting of 6 ng DNA, 1× LightScanner Master Mix, 1 μM each forward and reverse primer. Each well was topped with one drop of mineral oil. The PCR amplification protocol was 94 °C for 30 s, followed by 30 s at the appropriate annealing temperature (57 or 58 °C) and extension at 72 °C for 30 s for 40 cycles. Following a final melting step at 95 °C for 30 s, the samples were cooled to 4 °C until HRM analysis. Amplicon melting occurred on the LightScanner where samples were heated to 98 °C over a period of 8 min with default settings. Analysis was performed using the LightScanner^®^ Instrument & Analysis Software small amplicon genotyping module.

### GBS library construction and sequencing

GBS libraries were constructed following Ward et al. (2013) and Elshire et al. (2011). Briefly, 100 ng of genomic DNA per sample were digested with 4 U of *Ape*KI (New England Biolabs, Ipswich, MA, USA) and then ligated with T4 ligase to 1.8 ng of combined common and unique barcode adapters (Elshire et al. 2011). Annealed and quantitated unique barcode and common adapters were provided by the Buckler Lab for Maize Genetics and Diversity, Cornell University (Ithaca, NY, USA) and Clemson University (Clemson, SC, USA) (Supplementary Table 1).

The GBS libraries were submitted to the Oregon State University Center for Genome Research and Biocomputing core facilities (Corvallis, OR, USA) for quantitation using a Qubit^®^ fluorometer (Invitrogen, Carlsbad, CA, USA). The size distribution of the library was confirmed by checking 1000 pg of DNA with the Bioanalyzer 2100 HS-DNA chip (Agilent Technologies, Santa Clara, CA, USA). Libraries were diluted to 10 nM based on Qubit^®^ readings and quantitative PCR (qPCR) was used to quantify the diluted libraries. For each pooled library, 15.5 pM were loaded for single-end Illumina^®^ sequencing of 101 cycles with the HiSeq™ 2000 (Illumina, Inc.) and analyzed using the Version 3 cluster generation and sequencing kits (Illumina, Inc.).

The libraries were sequenced in three lanes at three different times. The first sequencing lane included 95 samples (91 progeny, and two replicated samples per parent). The second sequencing run included 88 samples (26 black raspberry including parents, grandparents, standards and progeny, and 62 unrelated strawberry samples). The third sequencing run included 64 samples (ORUS 3021-2 repeated 4 times, ORUS 4153-1 repeated 5 times and 55 progeny). Over all three runs, the parents ORUS 3021-2 and ORUS 4153-1 were sequenced at least twice in each lane. Forty-four progeny were sequenced more than once due to low initial quality and numbers of reads per individual.

### GBS SNP calling

Version 3.0 of the TASSEL GBS discovery software pipeline (Li et al. 2009) was used to call SNP loci using a repeat-masked version of the genome sequence. Three GBS runs representing 112 individuals as described above were analyzed simultaneously. Data were initially subjected to sequence and nucleotide read quality control using Trimmomatic (Bolger et al. 2014) (http://www.usadellab.org/cms/?page=trimmomatic) and were then analyzed with TASSEL.

### Genetic linkage map construction

All loci were converted into segregation codes for JoinMap^®^ v. 4.1 (Van Ooijen 2006). Loci were then organized into parental sets and subjected to the maximum likelihood (ML) mapping algorithm. Independence Likelihood of Odds (LOD) threshold of 5 was used for establishing the linkage groups (LG). All other settings were default. Five progeny (ORUS 4305-38, 39, 41, 59, and 65) were excluded based on incongruous SNP data occurring from 30 (ORUS 4305-39) to 90 (ORUS 4305-65) times. GBS data were not available for ORUS 4305-7, 19, 45, 54, 58, 75, 95, 97, 103, and 110 due to poor DNA quality. The consensus map of seven linkage groups was generated by combining the parental linkage maps of ORUS 3021-2 and ORUS 4153-1 using the regression algorithm of the mapping software JoinMap v. 4.1. Linkage map visualization was accomplished with MapChart 2.2 (Voorrips 2002).

The quality of genotype calls and of each map were evaluated with a graphical genotyping approach in Microsoft Excel (Redmond, WA, USA) as previously described (Bassil et al. 2015; Young and Tanksley 1989).

## Results

### Transferable markers

In total, 552 SSR markers from new and published sources were evaluated for the amplification of polymorphic PCR products in the parents and one progeny. Of these, 118 failed to amplify, 235 were homozygous in both parents or gave ambiguous results, 138 were heterozygous in both parents, 29 were heterozygous in ORUS 3021-2, and 32 were heterozygous in ORUS 4153-1 (Table 2).

A total of 30 primer pairs (SSR and HRM) for 28 anchor loci were assessed for the production of a polymorphic PCR product in the parents and six progeny of population ORUS 4305. Twelve of these loci were successfully mapped (Table 2).

Eighty HRM primer pairs (Bushakra et al. 2012) were evaluated for the amplification of polymorphic PCR products on the parents and 14 progeny. Of these 80 HRM primer pairs, 57 were monomorphic, 12 were unclear or had poor amplification, and 11 were evaluated in the full population. Three of these HRM markers were mapped successfully, two in ORUS 3021-2 and one in ORUS 4153-1 (Table 2). Out of 660 transferable markers evaluated, a total of 72 (11 %) were successfully mapped. BLAST analysis (Altschul et al. 1990) of the forward and reverse primer and nucleotide sequences (when available), allowed scaffold assignment of most mapped transferable markers (Supplementary Table 2).

### GBS SNP markers

The first sequencing run of 95 samples generated 596 K sequence clusters/mm^2^ (optimal density is 750–850 K clusters/mm^2^; MyIllumina Support Bulletin); the second and third sequencing runs were within the optimum range at 825 and 752 K clusters/mm^2^, respectively. These cluster densities provided raw reads ranging from approximately 165 million to 310 million. Over the three sequencing runs, 112 progeny and the two parents were sequenced to generate an average number of reads per individual of 3,105,333, with 20,317,182 (5.8 %) of reads unaligned. Default TASSEL filtering parameters using the parent information identified 57,238 SNP positions. Further filtering of the SNP data to remove those loci with more than 10 % missing data resulted in a data set of 7911 SNP loci, of which 3472 were monomorphic or ambiguous, 921 were heterozygous in both parents, 318 were heterozygous in ORUS 3021-2, and 326 were heterozygous in ORUS 4153-1 (Table 2).

### Linkage mapping

Of the five progeny excluded based on incongruous SNP data, ORUS 4305-65 showed obvious phenotypic differences from the rest of the population and may be the result of a pollen contamination; however, the other four progeny were not phenotypically different from the rest of the population. A total of 100 progeny were used to construct the seven linkage groups for the parental linkage maps, the characteristics of which are summarized in Table 3. To construct the linkage map for ORUS 3021-2, five GBS-generated SNP markers were removed for skewed segregation ratios, four were removed for creating double recombination events within a distance of 10 cM or less, and one was removed due to unsuccessful linkage phase determination. For ORUS 3021-2 (Supplementary Fig. 1) the resulting 274 markers comprising the seven LGs spanned 779.4 centiMorgans (cM) with an average distance of 2.9 cM between markers. RLG7 had the greatest number of markers (56), and was also the longest (134.5 cM) with an average distance of 2.4 cM between markers. RLG2 was the shortest at 84.1 cM, with an average distance of 2.8 cM between the 30 markers, and two gaps of 11.4 and 11.9 cM. The largest gap for the map of ORUS 3021-2 was 22.2 cM on RLG6. Of the 222 GBS SNP markers used for map construction, 200 (90 %) segregated as expected, either 1:1 or 1:2:1; two loci (1 %) varied from expected at a significance level of 0.01, 11 loci (5 %) varied from expected at a significance level of 0.05, and nine loci (4 %) varied from expected at a significance level of 0.1.

**Table 3:** Summary of genetic linkage maps for female parent ORUS 3021-2, male parent ORUS 4153-1, and the consensus map for F_1_ population ORUS 4305

	ORUS 3021-2 (female parent)					ORUS 4153-1 (male parent)					Consensus Map				
	Locus number	Number of transferable loci	cM	Average distance between loci in cM	Gaps over 10 cM	Locus number	Number of transferable loci	cM	Average distance between loci in cM	Gaps over 10 cM	Locus number	Number of transferable loci	cM	Average distance between loci in cM	Gaps over 10 cM
RLG1	29	8	99.5	3.4	2	23	6	101.7	4.4	3	39	9	77.5	2.0	0
RLG2	30	8	84.1	2.8	2	40	7	115.9	2.9	1	59	11	70.8	1.2	0
RLG3	44	5	113.6	2.6	1	33	4	102.9	3.1	1	67	7	73.2	1.1	0
RLG4	38	8	115.3	3.0	1	43	12	143.0	3.3	1	63	14	74.6	1.2	0
RLG5	45	10	134.0	3.0	1	43	4	127.8	3.0	2	64	10	79.1	1.2	0
RLG6	32	10	98.4	3.1	1	46	3	149.4	3.2	3	69	12	90.2	1.3	1
RLG7	56	3	134.5	2.4	0	64	6	151.4	2.4	3	77	7	81.0	1.1	1
**Total**	**274**	**52**	**779.4**	**2.9**		**292**	**42**	**892.1**	**3.2**		**438**	**70**	**546.4**	**1.3**	

Each *Rubus* linkage group (RLG) details the number of loci mapped, the number of loci that are transferable (either simple sequence repeat or high-resolution melting), the length of the linkage group in centiMorgans (cM), the average distance between each locus in cM, and the number of gaps larger than 10 cM

To construct the linkage map for ORUS 4153-1, 18 GBS-generated SNP markers were removed for skewed segregation ratios, 14 were removed for creating double recombination events within a distance of 10 cM or less, and one SSR marker was removed due to unsuccessful linkage phase determination. For ORUS 4153-1 (Supplementary Fig. 2) the resulting 292 markers comprising the seven LGs spanned 892.1 cM with an average distance of 3.2 cM between markers. RLG7 had the greatest number of markers (64) and was also the longest (151.4 cM) with an average distance of 2.4 cM between markers, and three gaps greater than 10 cM, the largest of which was 12.2 cM; RLG1 was the shortest at 101.7 cM with 23 markers, an average distance of 4.4 cM between markers, and three gaps greater than 10 cM, the largest of which was 14.8 cM. The largest gap for the map of ORUS 4153-1 was 14.8 cM at the end of RLG1. Of the 249 GBS SNP markers used for map construction, 230 (92 %) segregated as expected, either 1:1 or 1:2:1; a single locus (0.4 %) varied from expected at a significance level of 0.01, nine loci (4 %) varied from expected at a significance level of 0.05, and nine loci (4 %) varied from expected at a significance level of 0.1.

Transferable markers for the parental maps ranged from a low of three markers on ORUS 3021-2 RLG7 and 4153-1 RLG6 to a high of 12 on ORUS 4153-1 RLG4. A total of 72 transferable markers were mapped in this population. BLAST analysis of the transferable markers against the draft genome assembly allowed scaffold assignment for 65 of 72 markers (90 %) so that 356 scaffolds were represented.

**Fig. 2:**
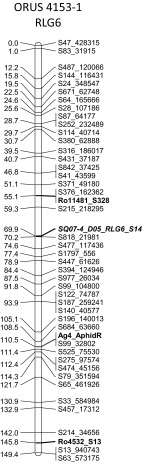
*Rubus* linkage group (RLG) 6 for black raspberry mapping population parent ORUS 4153-1. The morphological locus for *Ag*_*4*_ aphid resistance against the North American large raspberry aphid is shown in *blue bold font*. The linkage map is constructed of single-nucleotide polymorphic (SNP) loci generated by genotyping by sequencing (GBS) (prefaced with S) and simple sequence repeat (SSR) loci from various *Rubus* sources (prefaced with Ro, Ri, Rh, Ru, Rub, and SQ). Transferable loci are indicated in *bold font*; anchor loci for comparisons with other *Rubus* linkage maps are indicated in *bold italic font* (color figure online)

The phenotypic marker for aphid resistance, Ag4_AphidR, was located on RLG6 of the aphid-resistant parent ORUS 4153-1 and maps to the same location as S99_32802 (Fig. 2).

**Fig. 3:**
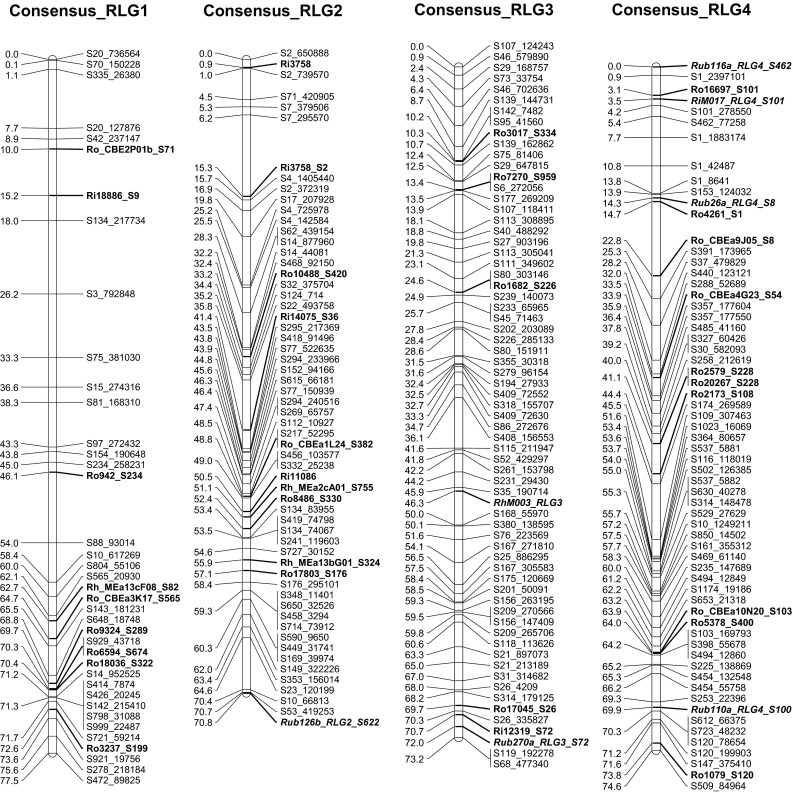

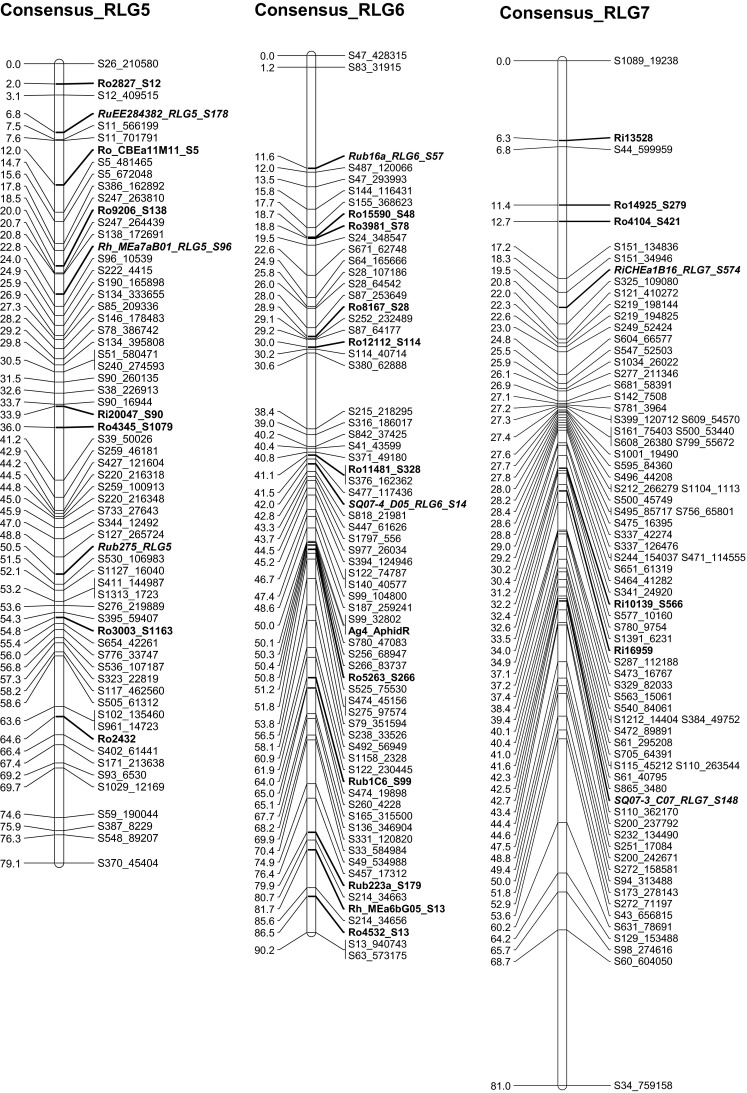
Consensus linkage map for population ORUS 4305. Each of the linkage groups consists of single-nucleotide polymorphic (SNP) loci generated by genotyping by sequencing (GBS) (prefaced with S) and simple sequence repeat (SSR) loci from various *Rubus* sources (prefaced with Ro, Ri, Rh, Ru, Rub, and SQ). Transferable loci are indicated in *bold font*; anchor loci for comparisons with other *Rubus* linkage maps are indicated in *bold italic font*. The morphological locus for *Ag*_*4*_ aphid resistance against the North American large raspberry aphid is shown in *blue bold font* (color figure online)

The seven consensus RLGs (Table 3; Fig. 3) assembled from merging the parental maps consisted of 438 markers spanning 546.4 cM with an average distance between markers of 1.3 cM. Consensus RLG6 was the longest (90.2 cM) with an average distance between the 69 markers of 1.3 cM, and one gap of 10.4 cM. Consensus RLG7 had the most markers (77) that spanned 81.0 cM with an average distance of 1.1 cM between markers. RLG2 was the shortest at 70.8 cM with an average distance between the 59 markers of 1.2 cM. The 12 anchor markers identified from the literature (Table 1; Supplementary Figs. 1, 2, markers in italics) allowed the positive identification of consensus RLG 2-7, with the last, RLG1, identified by default.

Thirteen of the 356 represented scaffolds (3.6 %) map to more than one linkage group (Table 4); 33 of the loci are SNPs and five are SSRs. Four scaffolds (S10, S26, S134, and S142) are represented by SNP loci on more than two linkage groups. Four scaffolds (S14, S71, S78, and S279) are represented by at least one SNP and a single SSR locus on more than one linkage group.

**Table 4:** Summary of the genomic scaffolds with loci on more than one *Rubus* linkage group (RLG)

Scaffold	RLG	Parent	SNP, SSR
S10	1, 2, 4	4153-1	3, 0
S14	1, 2	3021-2	2, 0
S14	2, 6	4153-1	1, 1
S21	3, 7	3021-2	2, 0
S26	3, 5, 7	3021-2	3, 0
S71	1, 2	3021-2	1, 1
S78	5, 6	3021-2	1, 1
S115	3, 7	3021-2	2, 0
S134	2, 5	3021-2	2, 0
S134	1, 2, 5	4153-1	3, 0
S142	1, 3	3021-2	2, 0
S142	3, 7	4153-1	2, 0
S161	7	3021-2	1, 0
S161	4	4153-1	1, 0
S279	3, 7	4153-1	1, 1
S380	3	3021-2	1, 0
S380	6	4153-1	1, 0
S472	1, 7	3021-2	2, 0

Each entry details the linkage group and the parental map on which the loci are found and the type of locus, either single-nucleotide polymorphic (SNP) or simple sequence repeat (SSR)

## Discussion

We present the first linkage map constructed from a pure black raspberry cross. The first attempt at genetic linkage mapping using SSR markers on an F_2_ generation of a black raspberry × red raspberry cross identified high homozygosity as well as severe segregation distortion and did not result in a linkage map (Lewers and Weber 2005). The linkage map constructed using non-anonymous DNA sequences for black raspberry selection 96395S1 comprises 29 markers spaced on average at intervals of 10 cM over six LG spanning 306 cM (Bushakra et al. 2012). The first published red raspberry map of ‘Glen Moy’ × ‘Latham’ consisted of 273 markers derived from amplified fragment length polymorphic and genomic-SSR markers and spanned 789 cM over nine LG (Graham et al. 2004). Over the next 6 years as more markers were developed and added, the improved ‘Glen Moy’ × ‘Latham’ map reported by Woodhead et al. (2010) consisted of 228 markers over seven LG spanning 840.3 cM with transferable markers present on each LG. Paterson et al. (2013) subsequently added gene-based markers to the linkage map constructed by Woodhead et al. (2010) by mining *Rubus* transcriptome and EST databases for candidate genes in the fruit volatiles pathway. The efficiency of marker generation used here is a vast improvement over previous marker development techniques in *Rubus*. The saturated consensus linkage map presented here spans 546.2 cM and is composed of 374 GBS-generated SNP markers and 68 transferable markers with an average of 1.3 cM between markers. The transferable markers are distributed among the LG and can be used for alignment to other *Rubus* maps. The scaffold assignment allows for future fine mapping, QTL analysis, and improved black raspberry genome assembly.

The reduced-representation sequencing accomplished with GBS has generally been used in crop plants with high levels of heterozygosity. For example, Poland et al. (2012) were able to map 20,000 and 34,000 GBS-generated SNP loci in wheat and barley reference linkage maps, respectively; Lu et al. (2013) performed GBS in tetraploid switchgrass (*Panicum virgatum*) and were able to map 88,217 SNP loci; Truong et al. (2012) used GBS to generate SNP in *Arabidopsis thaliana* and lettuce (*Lactuca sativa*) and were able to map 1200 and 1113 SNP loci, respectively; Russell et al. (2014) mapped 790 SNP loci in blackcurrant (*Ribes nigrum*). This is the first use of GBS on black raspberry, a crop of relatively low genetic diversity. Even with an average number of reads per individual of 3,105,333 over the three sequencing runs, only 1545 SNP loci were found that met criteria for linkage mapping and of those 399 were mapped successfully (Table 2). While this is a sufficient number of markers to develop a well-populated map, it, along with the low mapping success rate of transferable markers, illustrates the low level of heterozygosity found in black raspberry. In contrast, the linkage maps constructed of GBS-derived SNP and SSR markers for red raspberry parents ‘Heritage’ and ‘Tulameen’ comprise 4521 markers spaced on average at intervals of 0.1 cM over seven LG spanning 462.7 cM and 2391 markers spaced on average at intervals of 0.1 cM spanning 376.6 cM, respectively (Ward et al. 2013). While digestion by a more frequent restriction enzyme cutter for GBS may be a way to increase the number of SNP loci identified, this does not guarantee mapping success as segregation within the population is essential for linkage mapping.

Up to 97 % of the mapped scaffolds were placed on a single linkage group indicating high quality assembly of the draft genome. The 13 scaffolds that map to multiple LGs will need to be investigated further to assess whether these inconsistencies represent errors in the genome assembly; however, initial observations could indicate regions of high chromosome homology or possible regions of genome duplication especially between RLG3 and RLG7.

The placement of the aphid-resistance morphological marker representing gene *Ag*_*4*_ on RLG6 corresponds to the red raspberry genomic region found by Sargent et al. (2007) for *A*_*1*_. The only other aphid-resistance gene in *Rubus* that has been mapped is *A*_*10*_, which was found to be located on red raspberry RLG4 (Fernández-Fernández et al. 2013). *A*_*1*_ originated from the old red raspberry ‘Baumforth’s A’ and confers race-specific resistance to three biotypes of the European large raspberry aphid, *Amphorophora idaei* Börner (biotypes 1, 3 and the *A*_*10*_-breaking; McMenemy et al. 2009), but is ineffective against the North American species *A. agathonica*. Ag4_AphidR maps to the same position as SNP S99_32802, providing us with a clearly defined region on which to focus our future fine-mapping efforts and comparative mapping to red raspberry. This linkage map region is associated with many quantitative trait loci (QTL) having to do with resistance to aphids (Sargent et al. 2007), and fungal (Graham et al. 2006) and fungal-like (Graham et al. 2011) pathogens in red raspberry and we hope to use our linkage map to better understand the underlying reasons for these associations.

## Conclusions

We present here the first genetic linkage map of black raspberry comprised of GBS-generated SNP and transferable markers. The presence of SSR and HRM markers selected from the literature, along with the other transferable markers allowed us to positively identify all RLG as per Bushakra et al. (2012), and provide an opportunity to align all existing *Rubus* linkage maps. These maps will serve as a framework for anchoring scaffold sequences in the black raspberry draft genome sequence. Comparative mapping using the common markers and the draft genome sequence will be useful for aligning QTL among different species of *Rubus*. Future studies on the different sources of aphid resistance, including construction of densely populated linkage maps and cloning of loci associated with aphid resistance, will provide information on the loci and will result in the development of markers that can be used for marker-assisted breeding for aphid resistance in black raspberry.

### Author contribution statement

JMB Project Coordinator performed marker screening, selected anchor markers, ran and scored all markers, constructed the genetic linkage map, and wrote the manuscript. DWB developed a custom pipeline for bioinformatic analyses, and performed GBS SNP calling. MD developed the mapping population, short-read Ro and Ri primers, performed the initial marker screening, and phenotyped aphid-resistance in the mapping population. KJV and RVB assisted in GBS SNP calling and other bioinformatic analyses, BLAST analyses, and linkage mapping. BSG assisted in developing and performed the initial marker screening. JL PI on NIFA SCRI grant (project main funding) and contributed to manuscript writing. TCM PI on NIFA SCRI grant (project main funding) and contributed computational resources and bioinformatics analysis. CEF PI on NIFA SCRI grant (project main funding), helped assemble and phenotype the germplasm, develop the mapping population, and contributed to manuscript writing. Primary advisor for the phenotyping portion of the NIFA SCRI grant. NVB PI on NIFA SCRI grant (project main funding), helped analyze short-read sequencing results, develop and test molecular markers, and contributed to manuscript writing. Primary advisor for the genomics portion of the NIFA SCRI grant.

## Electronic supplementary material

Supplementary Fig. 1 A genetic linkage map of female parent ORUS 3021-2 constructed using the maximum likelihood algorithm of the mapping software JoinMap v. 4.1 Each of the linkage groups consists of single nucleotide polymorphic (SNP) loci generated by genotyping by sequencing (GBS) (prefaced with S) and simple sequence repeat (SSR) loci from various *Rubus* sources (prefaced with Ro, Ri, Rh, Ru, Rub, and SQ). Transferable loci are indicated in bold font; anchor loci for comparisons with other *Rubus* linkage maps are indicated in bold italic font (DOCX 57 kb)

Supplementary Fig. 1 A genetic linkage map of male parent ORUS 4153-1 constructed using the maximum likelihood algorithm of the mapping software JoinMap v. 4.1. Each of the linkage groups consists of single nucleotide polymorphic (SNP) loci generated by genotyping by sequencing (GBS) (prefaced with S) and simple sequence repeat (SSR) loci from various *Rubus* sources (prefaced with Ro, Ri, Rh, Ru, Rub, and SQ). Transferable loci are indicated in bold font; anchor loci for comparisons with other *Rubus* linkage maps are indicated in bold italic font (DOCX 42 kb)

Supplementary Table 1 Genotyping by sequencing unique barcode sequences. Well indicates the location on the 96-well plate, each barcode sequence is complemented. ^a^Each unique barcode sequence consists of two oligonucleotides 5′-ACACTCTTTCCCTACACGACGCTCTTCCGATCTxxxx and 5′-CWGyyyyAGATCGGAAGAGCGTCGTGTAGGGAAAGAGTGT, where ‘‘xxxx’’ and ‘‘yyyy’’ denote the barcode and barcode complement sequences, respectively. Common adapter sequences with an ApeKI-compatible sticky end are 5′-CWGAGATCGGAAGAGCGGTTCAGCAGGAATGCCGAG and 5′-CTCGGCATTCCTGCTGAACCGCTCTTCCGATCT (Elshire et al. 2011) (DOCX 14 kb)

Supplementary Table 2 Transferable locus basic linear alignment search tool (BLAST) results. Each primer sequence was compared to the black raspberry draft genome sequence using BLAST. Those loci for which a single hit was obtained were assigned a scaffold location. Those loci with both forward and reverse primers confirmed are indicated in bold font. Those loci with no confirmed scaffold location are in grey font (DOCX 47 kb)
