# Supplementary material for: A genetic linkage map of black raspberry (Rubus occidentalis) and the mapping of Ag4 conferring resistance to the aphid Amphorophora agathonica
Source: Theor Appl Genet. 2015 Jun 3;128(8):1631–46. doi: 10.1007/s00122-015-2541-x (PMC4477079; doi:10.1007/s00122-015-2541-x)
Supplement: Supplementary file 3 — Genotyping by sequencing unique barcode sequences. Well indicates the location on the 96-well plate, each barcode sequence is complemented. aEach unique barcode sequence consists of two oligonucleotides 5′-ACACTCTTTCCCTACACGACGCTCTTCCGATCTxxxx and 5′-CWGyyyyAGATCGGAAGAGCGTCGTGTAGGGAAAGAGTGT, where ‘‘xxxx’’ and ‘‘yyyy’’ denote the barcode and barcode complement sequences, respectively. Common adapter sequences with an ApeKI-compatible sticky end are 5′-CWGAGATCGGAAGAGCGGTTCAGCAGGAATGCCGAG and 5′-CTCGGCATTCCTGCTGAACCGCTCTTCCGATCT (Elshire et al. 2011) (DOCX 14 kb) [file 122_2015_2541_MOESM3_ESM.docx]

| Well | Barcode sequence^a^ | Well | Barcode sequence^a^ |
| --- | --- | --- | --- |
| A1 | CTCC | A7 | TGCGA |
| B1 | TTCTC | B7 | GGTTGT |
| C1 | GCTTA | C7 | ATGAAAC |
| D1 | AACGCCT | D7 | TAGGCCAT |
| E1 | AGGC | E7 | CCTAC |
| F1 | TCGTT | F7 | CTATTA |
| G1 | TGGCTA | G7 | CGGTAGA |
| H1 | TGCTGGA | H7 | CGTGTGGT |
| A2 | TGCA | A8 | CGAT |
| B2 | AGCCC | B8 | CCAGCT |
| C2 | CTTCCA | C8 | AAAAGTT |
| D2 | AATATGC | D8 | TGCAAGGA |
| E2 | GATC | E8 | GAGGA |
| F2 | ACCTAA | F8 | GCCAGT |
| G2 | ACGTGTT | G8 | CTACGGA |
| H2 | AACCGAGA | H8 | GCTGTGGA |
| A3 | ACTA | A9 | CGCTT |
| B3 | GTATT | B9 | TTCAGA |
| C3 | GAGATA | C9 | GAATTCA |
| D3 | ACGACTAC | D9 | TGGTACGT |
| E3 | TCAC | E9 | GGAAC |
| F3 | ATATGT | F9 | GGAAGA |
| G3 | ATTAATT | G9 | GCGGAAT |
| H3 | ACAGGGAA | H9 | GGATTGGT |
| A4 | CAGA | A10 | TCACC |
| B4 | CTGTA | B10 | TAGGAA |
| C4 | ATGCCT | C10 | GAACTTC |
| D4 | GGTGT | D10 | TCTCAGTC |
| E4 | AGGAT | E10 | GTCAA |
| F4 | ATCGTA | F10 | GTACTT |
| G4 | ATTGGAT | G10 | TAGCGGA |
| H4 | ACGTGGTA | H10 | GTGAGGGT |
| A5 | AACT | A11 | CTAGC |
| B5 | ACCGT | B11 | GCTCTA |
| C5 | TATTTTT | C11 | GGACCTA |
| D5 | TAGCATGC | D11 | CCGGATAT |
| E5 | ATTGA | E11 | TAATA |
| F5 | CATCGT | F11 | GTTGAA |
| G5 | CATAAGT | G11 | TCGAAGA |
| H5 | CCATGGGT | H11 | TATCGGGA |
| A6 | GCGT | A12 | ACAAA |
| B6 | GTAA | B12 | CCACAA |
| C6 | CTTGCTT | C12 | GTCGATT |
| D6 | AGTGGA | D12 | CGCCTTAT |
| E6 | CATCT | E12 | TACAT |
| F6 | CGCGGT | F12 | TAACGA |
| G6 | CGCTGAT | G12 | TCTGTGA |
| H6 | CGCGGAGA | H12 | TTCCTGGA |
