# Supplementary material for: A genetic linkage map of black raspberry (Rubus occidentalis) and the mapping of Ag4 conferring resistance to the aphid Amphorophora agathonica
Source: Theor Appl Genet. 2015 Jun 3;128(8):1631–46. doi: 10.1007/s00122-015-2541-x (PMC4477079; doi:10.1007/s00122-015-2541-x)
Supplement: Supplementary file 4 — Transferable locus basic linear alignment search tool (BLAST) results. Each primer sequence was compared to the black raspberry draft genome sequence using BLAST. Those loci for which a single hit was obtained were assigned a scaffold location. Those loci with both forward and reverse primers confirmed are indicated in bold font. Those loci with no confirmed scaffold location are in grey font (DOCX 47 kb) [file 122_2015_2541_MOESM4_ESM.docx]

| Forward primer | | | | | | | | Reverse primer | | | | | | | |
| --- | --- | --- | --- | --- | --- | --- | --- | --- | --- | --- | --- | --- | --- | --- | --- |
| SSR locus | Black raspberry genome scaffold | % Match | Length of match | Primer start pos. | Primer end pos. | Scaffold start pos. | Scaffold end pos. | SSR locus | Black raspberry genome scaffold | % Match | Length of match | Primer start pos. | Primer end pos. | Scaffold start pos. | Scaffold end pos. |
| ERubLR_SQ07_4_D05 | **S0014** | **100** | **19** | **19** | **37** | **1196475** | **1196493** | **ERubLSQ07_4_D05** | **S0014** | **100** | **20** | **1** | **20** | **1196717** | **1196698** |
| ERubLR_SQ07-3_C07 | **S0148** | **100** | **20** | **1** | **20** | **183671** | **183690** | **ERubLSQ07-3_C07** | **S0148** | **100** | **20** | **1** | **20** | **183799** | **183780** |
| RH_MEa0002cA01 | **S0755** | **100** | **20** | **1** | **20** | **19961** | **19980** | **RH_MEa0002cA01** | **S0755** | **100** | **20** | **1** | **20** | **20222** | **20203** |
| RH_MEa0002cA01 | S0293 | 100 | 20 | 1 | 20 | 134083 | 134102 |  |  |  |  |  |  |  |  |
| RH_MEa0002cA01 | S0184 | 100 | 20 | 1 | 20 | 115981 | 115962 |  |  |  |  |  |  |  |  |
| RH_MEa0002cA01 | S0141 | 100 | 18 | 1 | 18 | 50566 | 50583 |  |  |  |  |  |  |  |  |
| RH_MEa0002cA01 | S0134 | 100 | 17 | 1 | 17 | 246389 | 246405 |  |  |  |  |  |  |  |  |
| RH_MEa0006bG05 | **S0013** | **100** | **20** | **19** | **38** | **241465** | **241446** | **RH_MEa0006bG05** | **S0013** | **100** | **20** | **1** | **20** | **241184** | **241203** |
| RH_MEa0007aB01 | **S0096** | **100** | **20** | **1** | **20** | **223860** | **223879** | **RH_MEa0007aB01** | **S0096** | **100** | **20** | **1** | **20** | **223991** | **223972** |
| RH_MEa0007aB01 | S0035 | 100 | 18 | 1 | 18 | 571638 | 571621 |  |  |  |  |  |  |  |  |
| RH_MEa0013bG01 | **S0324** | **100** | **17** | **4** | **20** | **98632** | **98616** | **RH_MEa0013bG01** | **S0324** | **100** | **19** | **1** | **19** | **98406** | **98424** |
| RH_MEa0013cF08 | **S0082** | **100** | **20** | **1** | **20** | **388729** | **388710** | **RH_MEa0013cF08** | **S0082** | **100** | **20** | **1** | **20** | **388484** | **388503** |
| RhM003 | S0035 | 100 | 22 | 1 | 22 | 161037 | 161016 |  |  |  |  |  |  |  |  |
| RhM003 | S0015 | 100 | 17 | 4 | 20 | 611221 | 611237 |  |  |  |  |  |  |  |  |
| RI_CHEa0001B16 | **S0574** | **100** | **20** | **1** | **20** | **62981** | **62962** | **RI_CHEa0001B16** | **S0574** | **100** | **20** | **1** | **20** | **62900** | **62919** |
|  |  |  |  |  |  |  |  | RI_CHEa0001B16 | S0029 | 100 | 18 | 1 | 18 | 182814 | 182797 |
| Ri10139 | **S0566** | **100** | **19** | **4** | **22** | **9268** | **9250** | **Ri10139** | **S0566** | **100** | **19** | **4** | **22** | **8995** | **9013** |
| Ri10139 | S1439 | 100 | 19 | 4 | 22 | 1784 | 1802 | Ri10139 | S1091 | 100 | 22 | 1 | 22 | 7572 | 7593 |
| Ri10139 | S1029 | 100 | 19 | 4 | 22 | 14222 | 14240 | Ri10139 | S1015 | 100 | 22 | 1 | 22 | 34758 | 34737 |
| Ri10139 | S0861 | 100 | 19 | 4 | 22 | 54533 | 54551 | Ri10139 | S0716 | 100 | 22 | 1 | 22 | 71504 | 71483 |
| Ri10139 | S0311 | 100 | 19 | 4 | 22 | 798 | 816 | Ri10139 | S0715 | 100 | 22 | 1 | 22 | 17621 | 17642 |
| Ri10139 | S0241 | 100 | 19 | 4 | 22 | 17094 | 17076 | Ri10139 | S0278 | 100 | 22 | 1 | 22 | 22691 | 22670 |
| Ri10139 | S0076 | 100 | 19 | 4 | 22 | 399470 | 399452 | Ri10139 | S0206 | 100 | 22 | 1 | 22 | 85 | 106 |
| Ri10139 | S0027 | 100 | 19 | 4 | 22 | 593105 | 593123 | Ri10139 | S0080 | 100 | 22 | 1 | 22 | 532114 | 532135 |
|  |  |  |  |  |  |  |  | Ri10139 | S1107 | 100 | 19 | 4 | 22 | 8534 | 8516 |
|  |  |  |  |  |  |  |  | Ri10139 | S0536 | 100 | 19 | 4 | 22 | 9982 | 10000 |
| Ri11086 | S1309 | 100 | 20 | 1 | 20 | 5441 | 5422 | Ri11086 | S0184 | 100 | 20 | 3 | 22 | 71353 | 71372 |
| Ri11086 | S1188 | 100 | 20 | 1 | 20 | 13219 | 13238 | Ri11086 | S0379 | 100 | 19 | 3 | 21 | 143698 | 143680 |
| Ri11086 | S1087 | 100 | 20 | 1 | 20 | 10745 | 10764 |  |  |  |  |  |  |  |  |
| Ri11086 | S0854 | 100 | 20 | 1 | 20 | 53123 | 53142 |  |  |  |  |  |  |  |  |
| Ri11086 | S0493 | 100 | 20 | 1 | 20 | 105357 | 105376 |  |  |  |  |  |  |  |  |
| Ri11086 | S0444 | 100 | 20 | 1 | 20 | 77290 | 77309 |  |  |  |  |  |  |  |  |
| Ri11086 | S0436 | 100 | 20 | 1 | 20 | 27148 | 27167 |  |  |  |  |  |  |  |  |
| Ri11086 | S0315 | 100 | 20 | 1 | 20 | 146149 | 146130 |  |  |  |  |  |  |  |  |
| Ri11086 | S0264 | 100 | 20 | 1 | 20 | 263889 | 263870 |  |  |  |  |  |  |  |  |
| Ri11086 | S0238 | 100 | 20 | 1 | 20 | 21697 | 21716 |  |  |  |  |  |  |  |  |
| Ri12319 | **S0072** | **100** | **22** | **1** | **22** | **467392** | **467413** | **Ri12319** | **S0072** | **100** | **22** | **1** | **22** | **467675** | **467654** |
|  |  |  |  |  |  |  |  | Ri13528 | S1028 | 100 | 22 | 1 | 22 | 14383 | 14362 |
|  |  |  |  |  |  |  |  | Ri13528 | S0804 | 100 | 22 | 1 | 22 | 47996 | 48017 |
|  |  |  |  |  |  |  |  | Ri13528 | S0061 | 100 | 22 | 1 | 22 | 136802 | 136823 |
|  |  |  |  |  |  |  |  | Ri13528 | S0197 | 100 | 20 | 1 | 20 | 42995 | 43014 |
|  |  |  |  |  |  |  |  | Ri13528 | S0550 | 100 | 19 | 1 | 19 | 39108 | 39090 |
|  |  |  |  |  |  |  |  | Ri13528 | S1079 | 95.45 | 22 | 1 | 22 | 3711 | 3732 |
|  |  |  |  |  |  |  |  | Ri13528 | S0308 | 95.45 | 22 | 1 | 22 | 9703 | 9724 |
|  |  |  |  |  |  |  |  | Ri13528 | S0226 | 100 | 18 | 5 | 22 | 94982 | 94999 |
|  |  |  |  |  |  |  |  | Ri13528 | S0156 | 95.45 | 22 | 1 | 22 | 231720 | 231699 |
|  |  |  |  |  |  |  |  | Ri13528 | S0100 | 95.45 | 22 | 1 | 22 | 56574 | 56595 |
| Ri14075 | **S0036** | **100** | **19** | **1** | **19** | **273852** | **273870** | **Ri14075** | **S0036** | **100** | **22** | **1** | **22** | **274187** | **274166** |
|  |  |  |  |  |  |  |  | Ri16959 | S0367 | 100 | 22 | 1 | 22 | 19554 | 19575 |
|  |  |  |  |  |  |  |  | Ri16959 | S0712 | 95.45 | 22 | 1 | 22 | 57158 | 57137 |
|  |  |  |  |  |  |  |  | Ri16959 | S0715 | 100 | 17 | 1 | 17 | 17595 | 17611 |
| Ri18886 | **S0009** | **100** | **22** | **1** | **22** | **891389** | **891368** | **Ri18886** | **S0009** | **100** | **22** | **1** | **22** | **891098** | **891119** |
| Ri18886 | S0554 | 100 | 17 | 2 | 18 | 19590 | 19574 |  |  |  |  |  |  |  |  |
| Ri20047 | **S0090** | **100** | **23** | **1** | **23** | **331374** | **331396** | **Ri20047** | **S0090** | **100** | **20** | **1** | **20** | **331774** | **331755** |
| Ri20466 | S0058 | 95.45 | 22 | 1 | 22 | 659391 | 659370 | Ri20466 | S1410 | 100 | 22 | 1 | 22 | 7796 | 7817 |
| Ri20466 | S0654 | 100 | 17 | 2 | 18 | 30020 | 30036 | Ri20466 | S1293 | 100 | 22 | 1 | 22 | 14291 | 14312 |
| Ri20466 | S0080 | 100 | 17 | 1 | 17 | 532090 | 532106 | Ri20466 | S1129 | 100 | 22 | 1 | 22 | 19430 | 19409 |
|  |  |  |  |  |  |  |  | Ri20466 | S1047 | 100 | 22 | 1 | 22 | 24139 | 24160 |
|  |  |  |  |  |  |  |  | Ri20466 | S0997 | 100 | 22 | 1 | 22 | 1660 | 1639 |
|  |  |  |  |  |  |  |  | Ri20466 | S0864 | 100 | 22 | 1 | 22 | 24531 | 24510 |
|  |  |  |  |  |  |  |  | Ri20466 | S0818 | 100 | 22 | 1 | 22 | 46040 | 46061 |
|  |  |  |  |  |  |  |  | Ri20466 | S0818 | 100 | 17 | 6 | 22 | 1253 | 1237 |
|  |  |  |  |  |  |  |  | Ri20466 | S0743 | 100 | 22 | 1 | 22 | 17992 | 17971 |
|  |  |  |  |  |  |  |  | Ri20466 | S0716 | 100 | 22 | 1 | 22 | 71166 | 71187 |
|  |  |  |  |  |  |  |  | Ri20466 | S0716 | 100 | 22 | 1 | 22 | 16860 | 16839 |
|  |  |  |  |  |  |  |  | Ri20466 | S0716 | 95.24 | 21 | 1 | 21 | 16680 | 16660 |
|  |  |  |  |  |  |  |  | Ri20466 | S0715 | 100 | 22 | 1 | 22 | 17956 | 17935 |
| Ri3758 | S0002 | 100 | 22 | 1 | 22 | 780780 | 780759 |  |  |  |  |  |  |  |  |
| Ri5037 | **S0036** | **100** | **19** | **4** | **22** | **506600** | **506582** | **Ri5037** | **S0036** | **100** | **22** | **1** | **22** | **506285** | **506306** |
|  |  |  |  |  |  |  |  | Ri5037 | S0516 | 100 | 17 | 3 | 19 | 6905 | 6921 |
| RiM017 | **S0101** | **100** | **22** | **1** | **22** | **13714** | **13735** | **RiM017** | **S0101** | **100** | **21** | **2** | **22** | **13907** | **13887** |
| RiM017 | S0089 | 95.45 | 22 | 1 | 22 | 376222 | 376243 | RiM017 | S0089 | 100 | 19 | 2 | 20 | 376401 | 376383 |
|  |  |  |  |  |  |  |  | RiM017 | S0090 | 100 | 19 | 1 | 19 | 418869 | 418851 |
| Ro_CBEa0001L24 | **S0382** | **100** | **19** | **1** | **19** | **34320** | **34338** | **Ro_CBEa0001L24** | **S0382** | **100** | **20** | **1** | **20** | **34573** | **34554** |
|  |  |  |  |  |  |  |  | Ro_CBEa0001L24 | S0480 | 100 | 17 | 4 | 20 | 48767 | 48751 |
| Ro_CBEa0002P01b | **S0071** | **100** | **20** | **1** | **20** | **438579** | **438560** | **Ro_CBEa0002P01b** | **S0071** | **100** | **20** | **1** | **20** | **438371** | **438390** |
| Ro_CBEa0002P01b | S0350 | 100 | 18 | 1 | 18 | 160726 | 160709 |  |  |  |  |  |  |  |  |
| Ro_CBEa0003K17 | **S0565** | **100** | **20** | **1** | **20** | **58424** | **58443** | **Ro_CBEa0003K17** | **S0565** | **100** | **20** | **1** | **20** | **58711** | **58692** |
| Ro_CBEa0003K17 | S0254 | 100 | 18 | 3 | 20 | 203233 | 203250 |  |  |  |  |  |  |  |  |
| Ro_CBEa0003K17 | S0183 | 100 | 18 | 2 | 19 | 178455 | 178438 |  |  |  |  |  |  |  |  |
| Ro_CBEa0003K17 | S0297 | 100 | 17 | 2 | 18 | 197485 | 197469 |  |  |  |  |  |  |  |  |
| Ro_CBEa0004G23 | **S0054** | **100** | **20** | **1** | **20** | **505134** | **505115** | **Ro_CBEa0004G23** | **S0054** | **100** | **20** | **1** | **20** | **504653** | **504672** |
| Ro_CBEa0009J05 | **S0008** | **100** | **20** | **1** | **20** | **1260260** | **1260279** | **Ro_CBEa0009J05** | **S0008** | **100** | **20** | **1** | **20** | **1260534** | **1260515** |
| Ro_CBEa0010N20 | **S0103** | **100** | **19** | **2** | **20** | **161728** | **161746** | **Ro_CBEa0010N20** | **S0103** | **100** | **20** | **1** | **20** | **161822** | **161803** |
| Ro_CBEa0010N20 | S2129 | 100 | 17 | 4 | 20 | 247 | 231 |  |  |  |  |  |  |  |  |
| Ro_CBEa0010N20 | S1994 | 100 | 17 | 4 | 20 | 1015 | 1031 |  |  |  |  |  |  |  |  |
| Ro_CBEa0010N20 | S0478 | 100 | 17 | 4 | 20 | 116266 | 116250 |  |  |  |  |  |  |  |  |
| Ro_CBEa0011M11 | **S0005** | **100** | **20** | **1** | **20** | **168286** | **168267** | **Ro_CBEa0011M11** | **S0005** | **100** | **21** | **1** | **21** | **168065** | **168085** |
|  |  |  |  |  |  |  |  | Ro_CBEa0011M11 | S0014 | 100 | 17 | 3 | 19 | 847902 | 847918 |
| Ro10488 | **S0420** | **100** | **22** | **1** | **22** | **31906** | **31885** | **Ro10488** | **S0420** | **100** | **22** | **1** | **22** | **31803** | **31824** |
| Ro1079 | **S0120** | **100** | **22** | **1** | **22** | **48152** | **48131** | **Ro1079** | **S0120** | **95.45** | **22** | **1** | **22** | **47952** | **47973** |
|  |  |  |  |  |  |  |  | Ro1079 | S0222 | 100 | 17 | 6 | 22 | 296616 | 296600 |
|  |  |  |  |  |  |  |  | Ro1079 | S0022 | 100 | 17 | 6 | 22 | 752963 | 752947 |
| Ro11481 | **S0328** | **100** | **22** | **1** | **22** | **17397** | **17418** | **Ro11481** | **S0328** | **100** | **22** | **1** | **22** | **17534** | **17513** |
| Ro11481 | S0067 | 100 | 19 | 3 | 21 | 142257 | 142275 | Ro11481 | S0152 | 100 | 18 | 1 | 18 | 9197 | 9180 |
| Ro12112 | **S0114** | **100** | **22** | **1** | **22** | **354319** | **354298** | **Ro12112** | **S0114** | **100** | **22** | **1** | **22** | **354189** | **354210** |
| Ro12112 | S0640 | 100 | 17 | 5 | 21 | 84786 | 84802 |  |  |  |  |  |  |  |  |
| Ro12112 | S0162 | 100 | 17 | 3 | 19 | 1084 | 1068 |  |  |  |  |  |  |  |  |
| Ro12112 | S0004 | 100 | 17 | 4 | 20 | 637300 | 637316 |  |  |  |  |  |  |  |  |
| Ro14925 | **S0279** | **100** | **22** | **1** | **22** | **211567** | **211588** | **Ro14925** | **S0279** | **100** | **22** | **1** | **22** | **211660** | **211639** |
| Ro14925 | S1059 | 100 | 20 | 1 | 20 | 12735 | 12754 |  |  |  |  |  |  |  |  |
| Ro14925 | S0423 | 95.45 | 22 | 1 | 22 | 35442 | 35463 |  |  |  |  |  |  |  |  |
| Ro14925 | S0170 | 95.45 | 22 | 1 | 22 | 316972 | 316993 |  |  |  |  |  |  |  |  |
| Ro14925 | S0014 | 95.45 | 22 | 1 | 22 | 1081842 | 1081821 |  |  |  |  |  |  |  |  |
| Ro15590 | **S0048** | **100** | **22** | **1** | **22** | **146838** | **146817** | **Ro15590** | **S0048** | **100** | **22** | **1** | **22** | **146663** | **146684** |
| Ro16697 | **S0101** | **100** | **22** | **1** | **22** | **59158** | **59137** | **Ro16697** | **S0101** | **100** | **22** | **1** | **22** | **59023** | **59044** |
| Ro1682 | **S0226** | **95.45** | **22** | **1** | **22** | **229436** | **229415** | **Ro1682** | **S0226** | **100** | **22** | **1** | **22** | **229332** | **229353** |
|  |  |  |  |  |  |  |  | Ro1682 | S0727 | 100 | 18 | 1 | 18 | 54705 | 54688 |
|  |  |  |  |  |  |  |  | Ro1682 | S0129 | 95.45 | 22 | 1 | 22 | 89428 | 89407 |
|  |  |  |  |  |  |  |  | Ro1682 | S0338 | 100 | 17 | 2 | 18 | 25107 | 25123 |
| Ro17045 | **S0026** | **100** | **22** | **1** | **22** | **510809** | **510830** | **Ro17045** | **S0026** | **100** | **22** | **1** | **22** | **510955** | **510934** |
| Ro17045 | S0021 | 100 | 22 | 1 | 22 | 200344 | 200365 |  |  |  |  |  |  |  |  |
| Ro17045 | S0047 | 100 | 17 | 1 | 17 | 261917 | 261933 |  |  |  |  |  |  |  |  |
| Ro17803 | **S0176** | **100** | **22** | **1** | **22** | **336801** | **336780** | **Ro17803** | **S0176** | **100** | **22** | **1** | **22** | **336665** | **336686** |
|  |  |  |  |  |  |  |  | Ro18036 | S0322 | 100 | 22 | 1 | 22 | 189963 | 189984 |
| Ro20267 | **S0228** | **100** | **22** | **1** | **22** | **181065** | **181044** | **Ro20267** | **S0228** | **100** | **22** | **1** | **22** | **180927** | **180948** |
| Ro20267 | S0541 | 100 | 17 | 4 | 20 | 57503 | 57519 | Ro20267 | S0228 | 100 | 18 | 1 | 18 | 171406 | 171423 |
| Ro20267 | S0044 | 100 | 17 | 2 | 18 | 725095 | 725079 | Ro20267 | S0677 | 100 | 17 | 1 | 17 | 51084 | 51068 |
| Ro2173 | **S0108** | **100** | **22** | **1** | **22** | **219609** | **219588** | **Ro2173** | **S0108** | **100** | **22** | **1** | **22** | **219413** | **219434** |
| Ro2173 | S0529 | 100 | 22 | 1 | 22 | 45109 | 45130 | Ro2173 | S0108 | 100 | 19 | 1 | 19 | 221232 | 221214 |
| Ro2173 | S0505 | 100 | 22 | 1 | 22 | 13077 | 13098 |  |  |  |  |  |  |  |  |
| Ro2173 | S0320 | 100 | 22 | 1 | 22 | 57236 | 57215 |  |  |  |  |  |  |  |  |
| Ro2173 | S0110 | 100 | 22 | 1 | 22 | 269068 | 269089 |  |  |  |  |  |  |  |  |
| Ro2173 | S0100 | 100 | 22 | 1 | 22 | 221397 | 221376 |  |  |  |  |  |  |  |  |
| Ro2173 | S1722 | 100 | 20 | 1 | 20 | 2144 | 2163 |  |  |  |  |  |  |  |  |
| Ro2173 | S0490 | 100 | 20 | 1 | 20 | 130269 | 130288 |  |  |  |  |  |  |  |  |
| Ro2173 | S0225 | 100 | 20 | 1 | 20 | 208340 | 208359 |  |  |  |  |  |  |  |  |
| Ro2173 | S0001 | 100 | 20 | 1 | 20 | 880205 | 880186 |  |  |  |  |  |  |  |  |
| Ro2432 | S0163 | 95.45 | 22 | 1 | 22 | 51584 | 51605 |  |  |  |  |  |  |  |  |
| Ro2579 | **S0228** | **100** | **22** | **1** | **22** | **170568** | **170589** | **Ro2579** | **S0228** | **100** | **22** | **1** | **22** | **170729** | **170708** |
| Ro2827 | **S0012** | **100** | **22** | **1** | **22** | **823547** | **823526** | **Ro2827** | **S0012** | **100** | **22** | **1** | **22** | **823434** | **823455** |
| Ro3003 | **S1163** | **100** | **22** | **1** | **22** | **20812** | **20833** | **Ro3003** | **S1163** | **100** | **22** | **1** | **22** | **20945** | **20924** |
| Ro3017 | **S0334** | **100** | **22** | **1** | **22** | **126822** | **126801** | **Ro3017** | **S0334** | **100** | **22** | **1** | **22** | **126675** | **126696** |
| Ro3237 | **S0199** | **100** | **22** | **1** | **22** | **130127** | **130106** | **Ro3237** | **S0199** | **100** | **22** | **1** | **22** | **130013** | **130034** |
| Ro3981 | **S0078** | **100** | **22** | **1** | **22** | **268922** | **268901** | **Ro3981** | **S0078** | **100** | **22** | **1** | **22** | **268828** | **268849** |
|  |  |  |  |  |  |  |  | Ro3981 | S0066 | 100 | 17 | 2 | 18 | 300762 | 300778 |
|  |  |  |  |  |  |  |  | Ro3981 | S0052 | 100 | 17 | 2 | 18 | 432699 | 432715 |
| Ro4104 | **S0421** | **100** | **22** | **1** | **22** | **35988** | **36009** | **Ro4104** | **S0421** | **100** | **22** | **1** | **22** | **36151** | **36130** |
|  |  |  |  |  |  |  |  | Ro4104 | S0317 | 100 | 21 | 2 | 22 | 11406 | 11426 |
|  |  |  |  |  |  |  |  | Ro4104 | S0317 | 95.24 | 21 | 2 | 22 | 15793 | 15773 |
|  |  |  |  |  |  |  |  | Ro4104 | S0272 | 100 | 21 | 2 | 22 | 198585 | 198565 |
|  |  |  |  |  |  |  |  | Ro4104 | S0221 | 100 | 21 | 2 | 22 | 9688 | 9668 |
|  |  |  |  |  |  |  |  | Ro4104 | S0152 | 100 | 21 | 2 | 22 | 172941 | 172921 |
|  |  |  |  |  |  |  |  | Ro4104 | S0152 | 95.24 | 21 | 2 | 22 | 389259 | 389279 |
|  |  |  |  |  |  |  |  | Ro4104 | S0114 | 100 | 21 | 2 | 22 | 135225 | 135245 |
|  |  |  |  |  |  |  |  | Ro4104 | S0114 | 95.24 | 21 | 2 | 22 | 432191 | 432171 |
|  |  |  |  |  |  |  |  | Ro4104 | S0062 | 100 | 21 | 2 | 22 | 643047 | 643067 |
|  |  |  |  |  |  |  |  | Ro4104 | S0062 | 95.24 | 21 | 2 | 22 | 643013 | 642993 |
|  |  |  |  |  |  |  |  | Ro4104 | S0014 | 100 | 21 | 2 | 22 | 931026 | 931006 |
|  |  |  |  |  |  |  |  | Ro4104 | S0014 | 95.24 | 21 | 2 | 22 | 931060 | 931080 |
|  |  |  |  |  |  |  |  | Ro4104 | S0003 | 100 | 21 | 2 | 22 | 2358348 | 2358368 |
|  |  |  |  |  |  |  |  | Ro4104 | S0003 | 95.24 | 21 | 2 | 22 | 552227 | 552247 |
|  |  |  |  |  |  |  |  | Ro4104 | S0166 | 100 | 20 | 3 | 22 | 363442 | 363461 |
| Ro4261 | **S0001** | **100** | **22** | **1** | **22** | **887965** | **887944** | **Ro4261** | **S0001** | **100** | **22** | **1** | **22** | **887772** | **887793** |
| Ro4261 | S0001 | 100 | 21 | 2 | 22 | 884044 | 884024 |  |  |  |  |  |  |  |  |
| Ro4345 | **S1079** | **100** | **22** | **1** | **22** | **18554** | **18575** | **Ro4345** | **S1079** | **100** | **22** | **1** | **22** | **18653** | **18632** |
| Ro4345 | S0038 | 95.24 | 21 | 1 | 21 | 807646 | 807626 |  |  |  |  |  |  |  |  |
| Ro4532 | **S0013** | **100** | **22** | **1** | **22** | **384679** | **384700** | **Ro4532** | **S0013** | **100** | **22** | **1** | **22** | **384867** | **384846** |
|  |  |  |  |  |  |  |  | Ro4532 | S0078 | 100 | 17 | 4 | 20 | 473284 | 473300 |
|  |  |  |  |  |  |  |  | Ro4532 | S0078 | 100 | 17 | 4 | 20 | 442977 | 442993 |
| Ro5263 | **S0266** | **100** | **22** | **1** | **22** | **42344** | **42323** | **Ro5263** | **S0266** | **100** | **22** | **1** | **22** | **42163** | **42184** |
|  |  |  |  |  |  |  |  | Ro5263 | S0527 | 95.24 | 21 | 1 | 21 | 72469 | 72489 |
| Ro5378 | **S0400** | **100** | **22** | **1** | **22** | **135908** | **135929** | **Ro5378** | **S0400** | **100** | **22** | **1** | **22** | **136105** | **136084** |
|  |  |  |  |  |  |  |  | Ro5378 | S0624 | 100 | 17 | 6 | 22 | 64466 | 64482 |
|  |  |  |  |  |  |  |  | Ro5378 | S0319 | 100 | 17 | 6 | 22 | 26891 | 26907 |
| Ro6594 | **S0674** | **100** | **22** | **1** | **22** | **62977** | **62956** | **Ro6594** | **S0674** | **100** | **22** | **1** | **22** | **62828** | **62849** |
| Ro7270 | **S0959** | **95.45** | **22** | **1** | **22** | **23309** | **23288** | **Ro7270** | **S0959** | **100** | **22** | **1** | **22** | **23150** | **23171** |
|  |  |  |  |  |  |  |  | Ro7270 | S0233 | 100 | 17 | 5 | 21 | 192612 | 192596 |
|  |  |  |  |  |  |  |  | Ro7270 | S0055 | 100 | 17 | 5 | 21 | 189602 | 189586 |
| Ro8167 | **S0028** | **100** | **22** | **1** | **22** | **360836** | **360857** | **Ro8167** | **S0028** | **100** | **22** | **1** | **22** | **360913** | **360892** |
| Ro8167 | S0273 | 100 | 17 | 1 | 17 | 10315 | 10331 |  |  |  |  |  |  |  |  |
| Ro8167 | S0056 | 95.24 | 21 | 2 | 22 | 373148 | 373168 |  |  |  |  |  |  |  |  |
| Ro8486 | **S0330** | **100** | **22** | **1** | **22** | **190871** | **190850** | **Ro8486** | **S0330** | **100** | **22** | **1** | **22** | **190714** | **190735** |
|  |  |  |  |  |  |  |  | Ro8486 | S0565 | 100 | 17 | 5 | 21 | 21755 | 21739 |
| Ro9206 | **S0138** | **100** | **22** | **1** | **22** | **306236** | **306257** | **Ro9206** | **S0138** | **100** | **22** | **1** | **22** | **306351** | **306330** |
| Ro9206 | S0351 | 100 | 20 | 3 | 22 | 44957 | 44976 |  |  |  |  |  |  |  |  |
| Ro9206 | S0152 | 100 | 20 | 3 | 22 | 50186 | 50167 |  |  |  |  |  |  |  |  |
| Ro9324 | **S0289** | **100** | **22** | **1** | **22** | **124243** | **124264** | **Ro9324** | **S0289** | **100** | **22** | **1** | **22** | **124377** | **124356** |
| Ro9324 | S0289 | 100 | 17 | 5 | 21 | 125161 | 125145 |  |  |  |  |  |  |  |  |
| Ro942 | **S0234** | **100** | **22** | **1** | **22** | **110679** | **110658** | **Ro942** | **S0234** | **100** | **22** | **1** | **22** | **110511** | **110532** |
| Ru_EE284382 | **S0178** | **100** | **20** | **1** | **20** | **232362** | **232381** | **Ru_EE284382** | **S0178** | **100** | **20** | **1** | **20** | **232539** | **232520** |
|  |  |  |  |  |  |  |  | Ru_EE284382 | S0155 | 100 | 17 | 3 | 19 | 72082 | 72066 |
|  |  |  |  |  |  |  |  | Rub1c6 | S0099 | 100 | 23 | 3 | 25 | 289910 | 289932 |
| Rubus110a | **S0100** | **100** | **23** | **1** | **23** | **271620** | **271642** | **Rubus110a** | **S0100** | **100** | **20** | **1** | **20** | **271803** | **271784** |
| Rubus110a | S2090 | 100 | 18 | 4 | 21 | 148 | 131 |  |  |  |  |  |  |  |  |
| Rubus110a | S1323 | 100 | 18 | 4 | 21 | 1843 | 1860 |  |  |  |  |  |  |  |  |
| Rubus110a | S1187 | 100 | 18 | 4 | 21 | 2209 | 2192 |  |  |  |  |  |  |  |  |
| Rubus110a | S1155 | 100 | 18 | 4 | 21 | 18174 | 18157 |  |  |  |  |  |  |  |  |
| Rubus110a | S1128 | 100 | 18 | 4 | 21 | 15157 | 15174 |  |  |  |  |  |  |  |  |
| Rubus110a | S1097 | 100 | 18 | 4 | 21 | 23998 | 23981 |  |  |  |  |  |  |  |  |
| Rubus110a | S0992 | 100 | 18 | 4 | 21 | 33898 | 33881 |  |  |  |  |  |  |  |  |
| Rubus110a | S0782 | 100 | 18 | 4 | 21 | 52934 | 52917 |  |  |  |  |  |  |  |  |
| Rubus110a | S0777 | 100 | 18 | 4 | 21 | 53869 | 53886 |  |  |  |  |  |  |  |  |
| Rubus110a | S0777 | 100 | 18 | 4 | 21 | 48502 | 48519 |  |  |  |  |  |  |  |  |
|  |  |  |  |  |  |  |  | Rubus116a | S0462 | 100 | 20 | 1 | 20 | 49057 | 49076 |
| Rubus126b | **S0622** | **100** | **22** | **1** | **22** | **59101** | **59122** | **Rubus126b** | **S0622** | **100** | **21** | **1** | **21** | **59235** | **59215** |
| Rubus16a | S0057 | 100 | 17 | 4 | 20 | 431817 | 431833 |  |  |  |  |  |  |  |  |
| Rubus223a | **S0179** | **100** | **24** | **1** | **24** | **202081** | **202058** | **Rubus223a** | **S0179** | **100** | **20** | **1** | **20** | **201944** | **201963** |
| Rubus223a | S0023 | 100 | 17 | 5 | 21 | 595111 | 595095 |  |  |  |  |  |  |  |  |
| Rubus26a | S0008 | 100 | 20 | 1 | 20 | 179650 | 179631 |  |  |  |  |  |  |  |  |
|  |  |  |  |  |  |  |  | Rubus256e | S0362 | 100 | 20 | 1 | 20 | 16693 | 16712 |
| Rubus270a | **S0072** | **100** | **20** | **1** | **20** | **495079** | **495060** | **Rubus270a** | **S0072** | **100** | **20** | **1** | **20** | **494909** | **494928** |
| Rubus270a | S1013 | 100 | 17 | 3 | 19 | 360 | 344 |  |  |  |  |  |  |  |  |
| Rubus270a | S0331 | 100 | 17 | 3 | 19 | 174282 | 174266 |  |  |  |  |  |  |  |  |
| Rubus270a | S0134 | 100 | 17 | 3 | 19 | 411189 | 411205 |  |  |  |  |  |  |  |  |
| Rubus270a | S0134 | 100 | 17 | 3 | 19 | 408217 | 408233 |  |  |  |  |  |  |  |  |
|  |  |  |  |  |  |  |  | Rubus275 | S0126 | 100 | 20 | 1 | 20 | 128240 | 128259 |
|  |  |  |  |  |  |  |  | Rubus275 | S0085 | 100 | 17 | 3 | 19 | 141722 | 141738 |
