# Supplementary material for: A genetic linkage map of black raspberry (Rubus occidentalis) and the mapping of Ag4 conferring resistance to the aphid Amphorophora agathonica
Source: Theor Appl Genet. 2015 Jun 3;128(8):1631–46. doi: 10.1007/s00122-015-2541-x (PMC4477079; doi:10.1007/s00122-015-2541-x)
Supplement: Supplementary file 3 [file 122_2015_2541_MOESM1_ESM.docx]

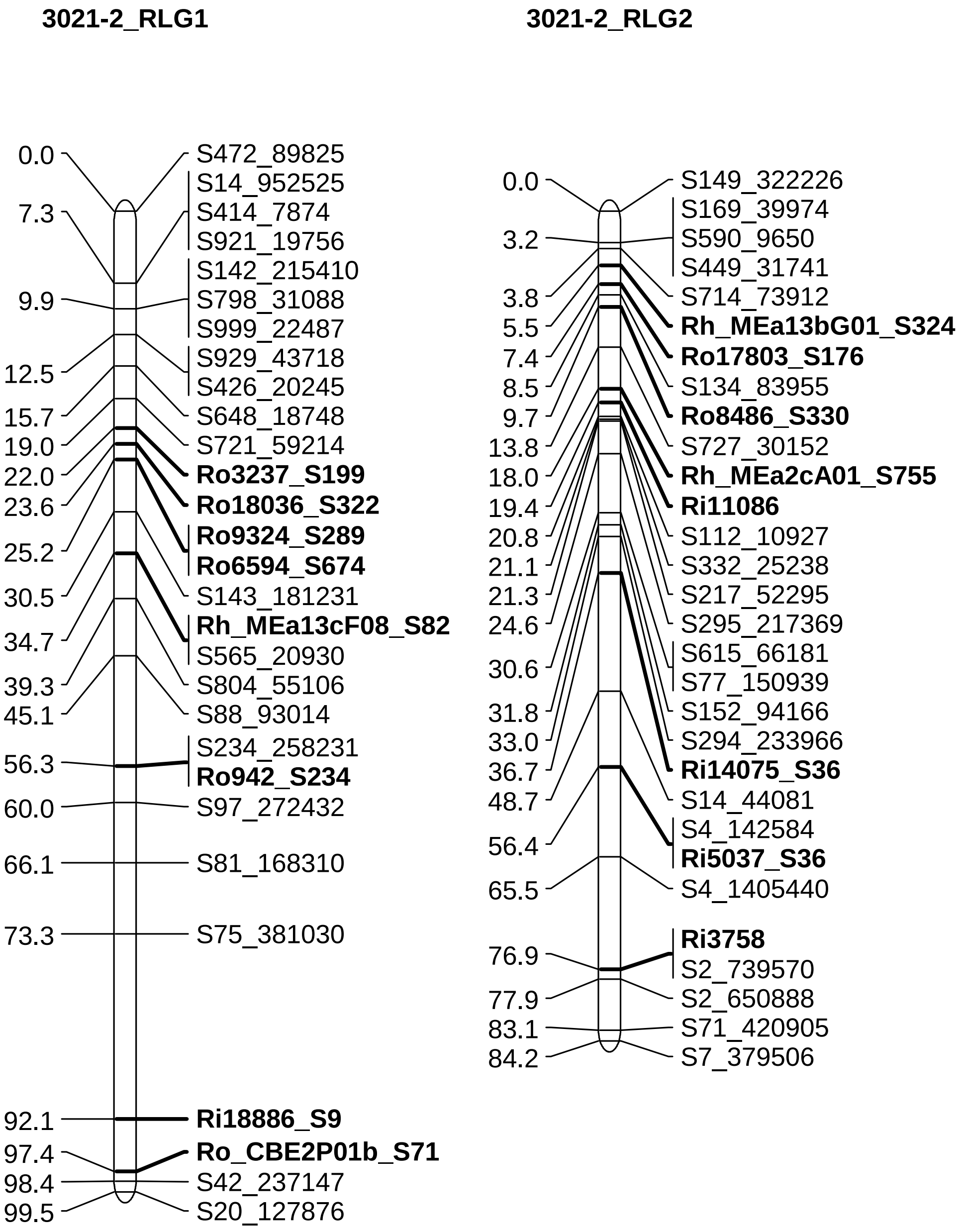
3021-2_RLG1
0.0 S472_89825
S14_952525
7.3 S414_7874
S921_19756
S142_215410
9.9 S798_31088
S999_22487
12.5 S929_43718
S426_20245
15.7 S648_18748
19.0 S721_59214
22.0 Ro3237_S199
23.6 Ro18036_S322
Ro9324_S289
25.2 Ro6594_S674
30.5 S143_181231
34.7 Rh_MEa13cF08_S82
S565_20930
39.3 S804_55106
45.1 S88_93014
S234_258231
56.3 Ro942_S234
60.0 S97_272432
66.1 S81_168310
73.3 S75_381030
92.1 Ri18886_S9
97.4 Ro_CBE2P01b_S71
98.4 S42_237147
99.5 S20_127876
3021-2_RLG2
0.0 S149_322226
S169_39974
3.2 S590_9650
S449_31741
3.8 S714_73912
5.5 Rh_MEa13bG01_S324
7.4 Ro17803_S176
8.5 S134_83955
9.7 Ro8486_S330
13.8 S727_30152
18.0 Rh_MEa2cA01_S755
19.4 Ri11086
20.8 S112_10927
21.1 S332_25238
21.3 S217_52295
24.6 S295_217369
S615_66181
30.6 S77_150939
31.8 S152_94166
33.0 S294_233966
36.7 Ri14075_S36
48.7 S14_44081
S4_142584
56.4 Ri5037_S36
65.5 S4_1405440
Ri3758
76.9 S2_739570
77.9 S2_650888
83.1 S71_420905
84.2 S7_379506

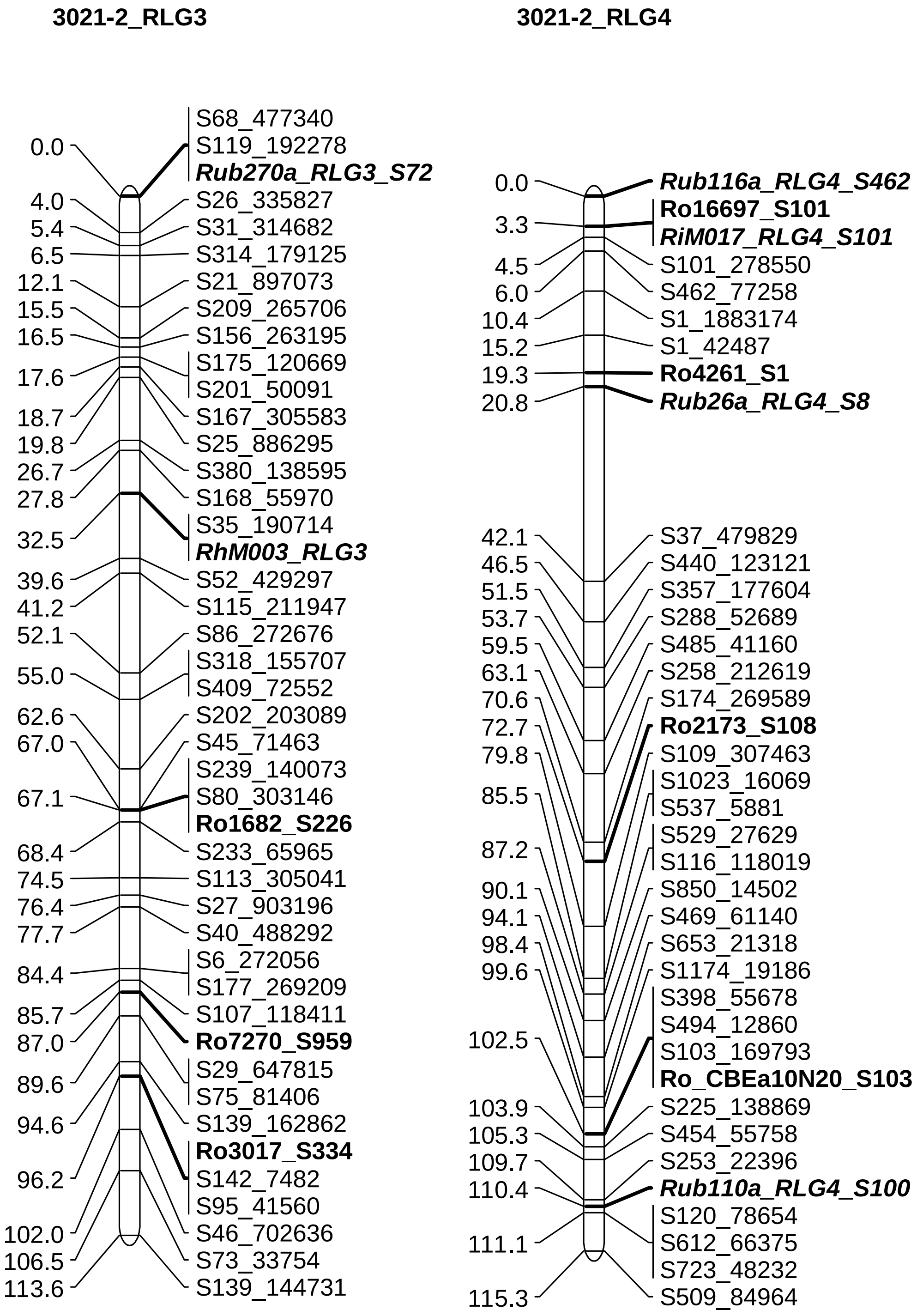
3021-2_RLG3
0.0 S68_477340
S119_192278
Rub270a_RLG3_S72
4.0 S26_335827
5.4 S31_314682
6.5 S314_179125
12.1 S21_897073
15.5 S209_265706
16.5 S156_263195
17.6 S175_120669
S201_50091
18.7 S167_305583
19.8 S25_886295
26.7 S380_138595
27.8 S168_55970
32.5 S35_190714
RhM003_RLG3
39.6 S52_429297
41.2 S115_211947
52.1 S86_272676
55.0 S318_155707
S409_72552
62.6 S202_203089
67.0 S45_71463
67.1 S239_140073
S80_303146
Ro1682_S226
68.4 S233_65965
74.5 S113_305041
76.4 S27_903196
77.7 S40_488292
84.4 S6_272056
S177_269209
85.7 S107_118411
87.0 Ro7270_S959
89.6 S29_647815
S75_81406
94.6 S139_162862
96.2 Ro3017_S334
S142_7482
S95_41560
102.0 S46_702636
106.5 S73_33754
113.6 S139_144731
3021-2_RLG4
0.0 Rub116a_RLG4_S462
3.3 Ro16697_S101
RiM017_RLG4_S101
4.5 S101_278550
6.0 S462_77258
10.4 S1_1883174
15.2 S1_42487
19.3 Ro4261_S1
20.8 Rub26a_RLG4_S8
42.1 S37_479829
46.5 S440_123121
51.5 S357_177604
53.7 S288_52689
59.5 S485_41160
63.1 S258_212619
70.6 S174_269589
72.7 Ro2173_S108
79.8 S109_307463
85.5 S1023_16069
S537_5881
87.2 S529_27629
S116_118019
90.1 S850_14502
94.1 S469_61140
98.4 S653_21318
99.6 S1174_19186
102.5 S398_55678
S494_12860
S103_169793
Ro_CBEa10N20_S103
103.9 S225_138869
105.3 S454_55758
109.7 S253_22396
110.4 Rub110a_RLG4_S100
111.1 S120_78654
S612_66375
S723_48232
115.3 S509_84964

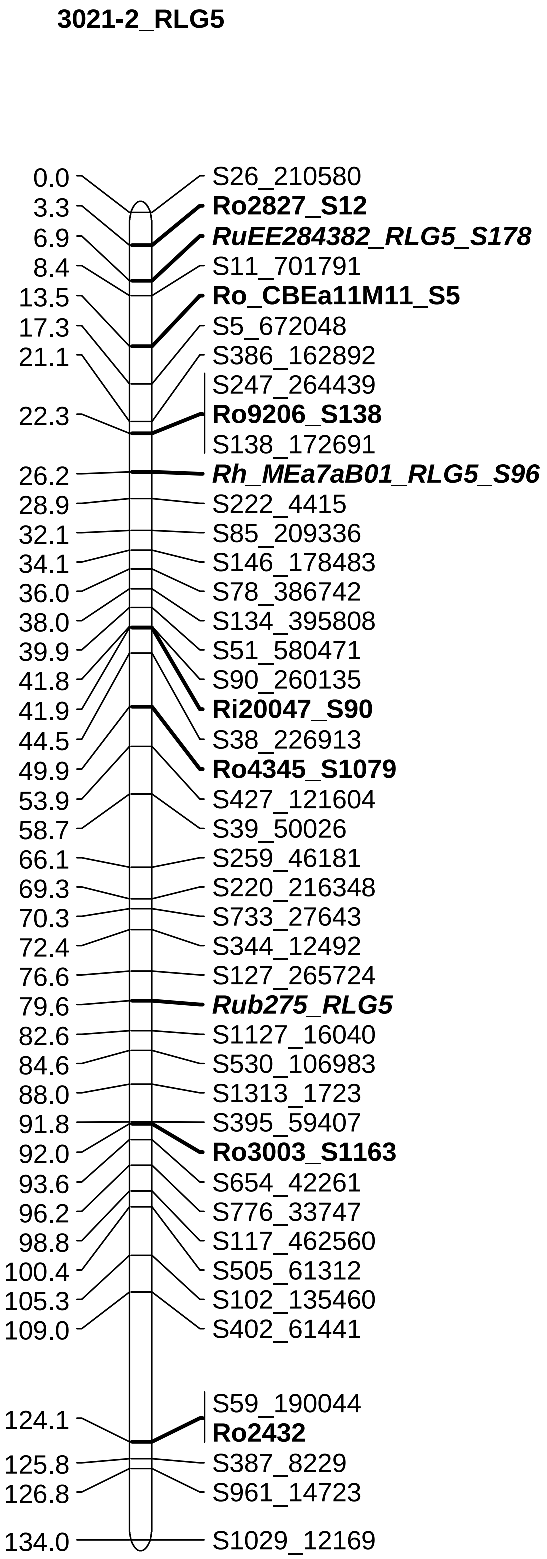
3021-2_RLG5
0.0 S26_210580
3.3 Ro2827_S12
6.9 RuEE284382_RLG5_S178
8.4 S11_701791
13.5 Ro_CBEa11M11_S5
17.3 S5_672048
21.1 S386_162892
22.3 S247_264439 Ro9206_S138 S138_172691
26.2 Rh_MEa7aB01_RLG5_S96
28.9 S222_4415
32.1 S85_209336
34.1 S146_178483
36.0 S78_386742
38.0 S134_395808
39.9 S51_580471
41.8 S90_260135
41.9 Ri20047_S90
44.5 S38_226913
49.9 Ro4345_S1079
53.9 S427_121604
58.7 S39_50026
66.1 S259_46181
69.3 S220_216348
70.3 S733_27643
72.4 S344_12492
76.6 S127_265724
79.6 Rub275_RLG5
82.6 S1127_16040
84.6 S530_106983
88.0 S1313_1723
91.8 S395_59407
92.0 Ro3003_S1163
93.6 S654_42261
96.2 S776_33747
98.8 S117_462560
100.4 S505_61312
105.3 S102_135460
109.0 S402_61441
124.1 S59_190044 Ro2432
125.8 S387_8229
126.8 S961_14723
134.0 S1029_12169
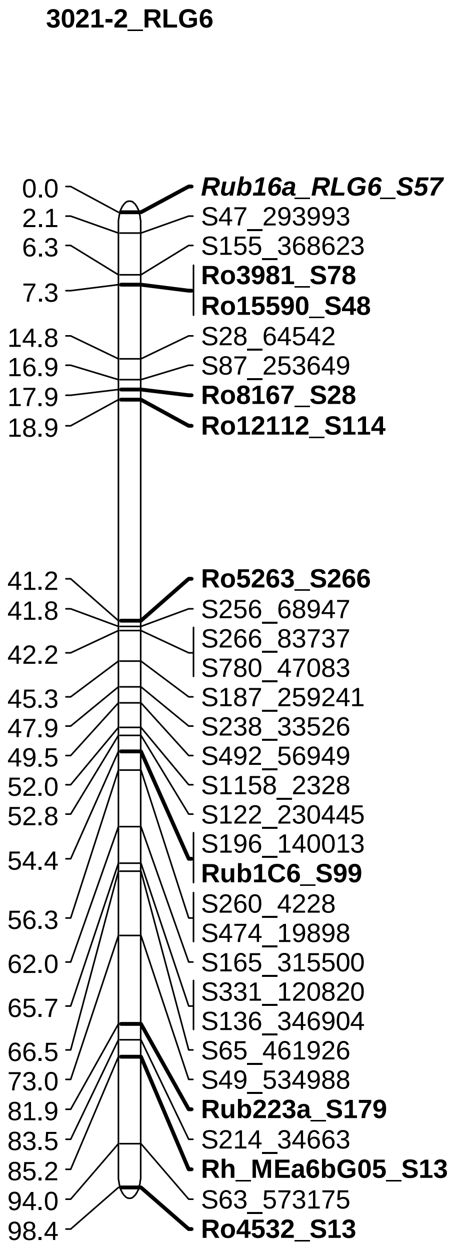
3021-2_RLG6
0.0 Rub16a_RLG6_S57
2.1 S47_293993
6.3 S155_368623
7.3 Ro3981_S78 Ro15590_S48
14.8 S28_64542
16.9 S87_253649
17.9 Ro8167_S28
18.9 Ro12112_S114
41.2 Ro5263_S266
41.8 S256_68947
42.2 S266_83737 S780_47083
45.3 S187_259241
47.9 S238_33526
49.5 S492_56949
52.0 S1158_2328
52.8 S122_230445
54.4 S196_140013 Rub1C6_S99
56.3 S260_4228 S474_19898
62.0 S165_315500
65.7 S331_120820 S136_346904
66.5 S65_461926
73.0 S49_534988
81.9 Rub223a_S179
83.5 S214_34663
85.2 Rh_MEa6bG05_S13
94.0 S63_573175
98.4 Ro4532_S13

3021-2_RLG7

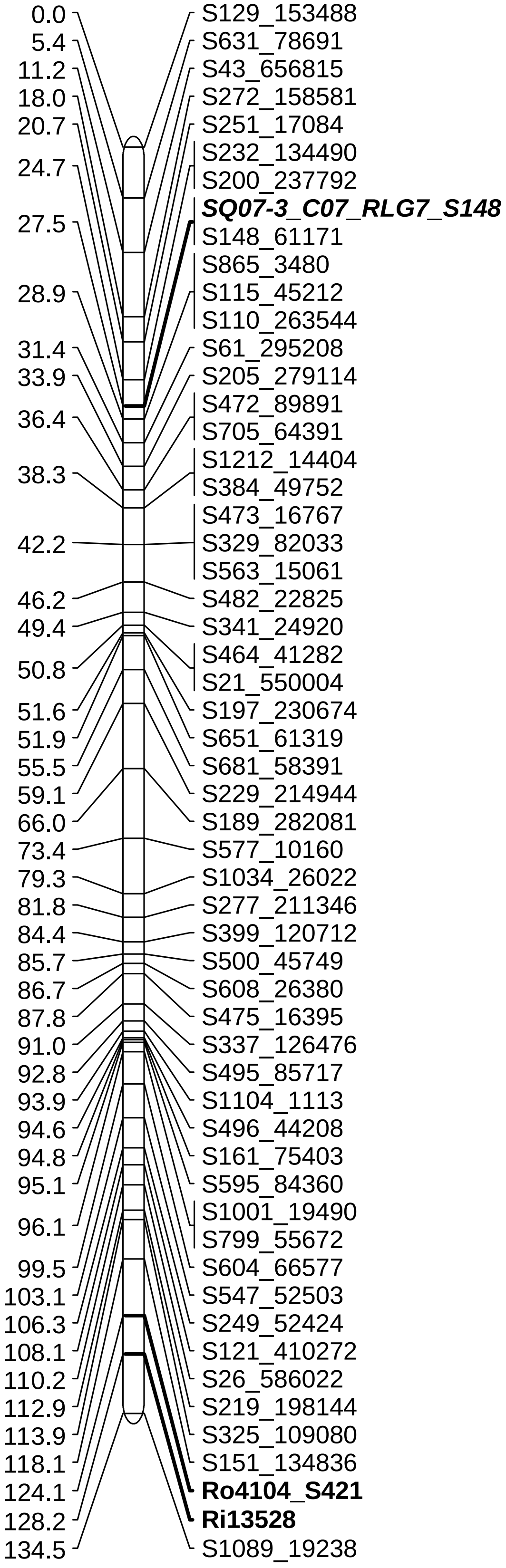
0.0
5.4
11.2
18.0
20.7
24.7
27.5
28.9
31.4
33.9
36.4
38.3
42.2
46.2
49.4
50.8
51.6
51.9
55.5
59.1
66.0
73.4
79.3
81.8
84.4
85.7
86.7
87.8
91.0
92.8
93.9
94.6
94.8
95.1
96.1
99.5
103.1
106.3
108.1
110.2
112.9
113.9
118.1
124.1
128.2
134.5
S129_153488
S631_78691
S43_656815
S272_158581
S251_17084
S232_134490
S200_237792
SQ07-3_C07_RLG7_S148
S148_61171
S865_3480
S115_45212
S110_263544
S61_295208
S205_279114
S472_89891
S705_64391
S1212_14404
S384_49752
S473_16767
S329_82033
S563_15061
S482_22825
S341_24920
S464_41282
S21_550004
S197_230674
S651_61319
S681_58391
S229_214944
S189_282081
S577_10160
S1034_26022
S277_211346
S399_120712
S500_45749
S608_26380
S475_16395
S337_126476
S495_85717
S1104_1113
S496_44208
S161_75403
S595_84360
S1001_19490
S799_55672
S604_66577
S547_52503
S249_52424
S121_410272
S26_586022
S219_198144
S325_109080
S151_134836
Ro4104_S421
Ri13528
S1089_19238
