# Supplementary material for: A genetic linkage map of black raspberry (Rubus occidentalis) and the mapping of Ag4 conferring resistance to the aphid Amphorophora agathonica
Source: Theor Appl Genet. 2015 Jun 3;128(8):1631–46. doi: 10.1007/s00122-015-2541-x (PMC4477079; doi:10.1007/s00122-015-2541-x)
Supplement: Supplementary file 4 [file 122_2015_2541_MOESM2_ESM.docx]

4153-1_RLG1

4153-1_RLG2

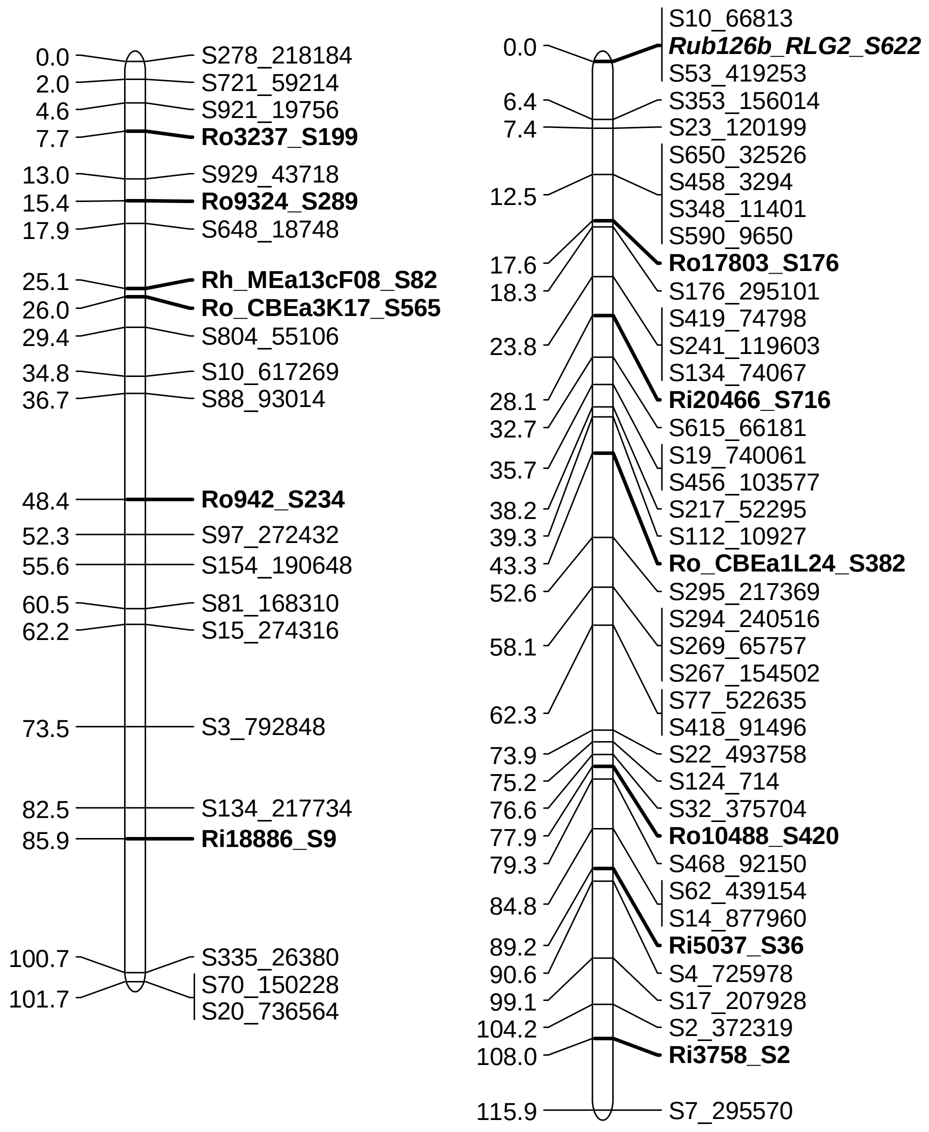

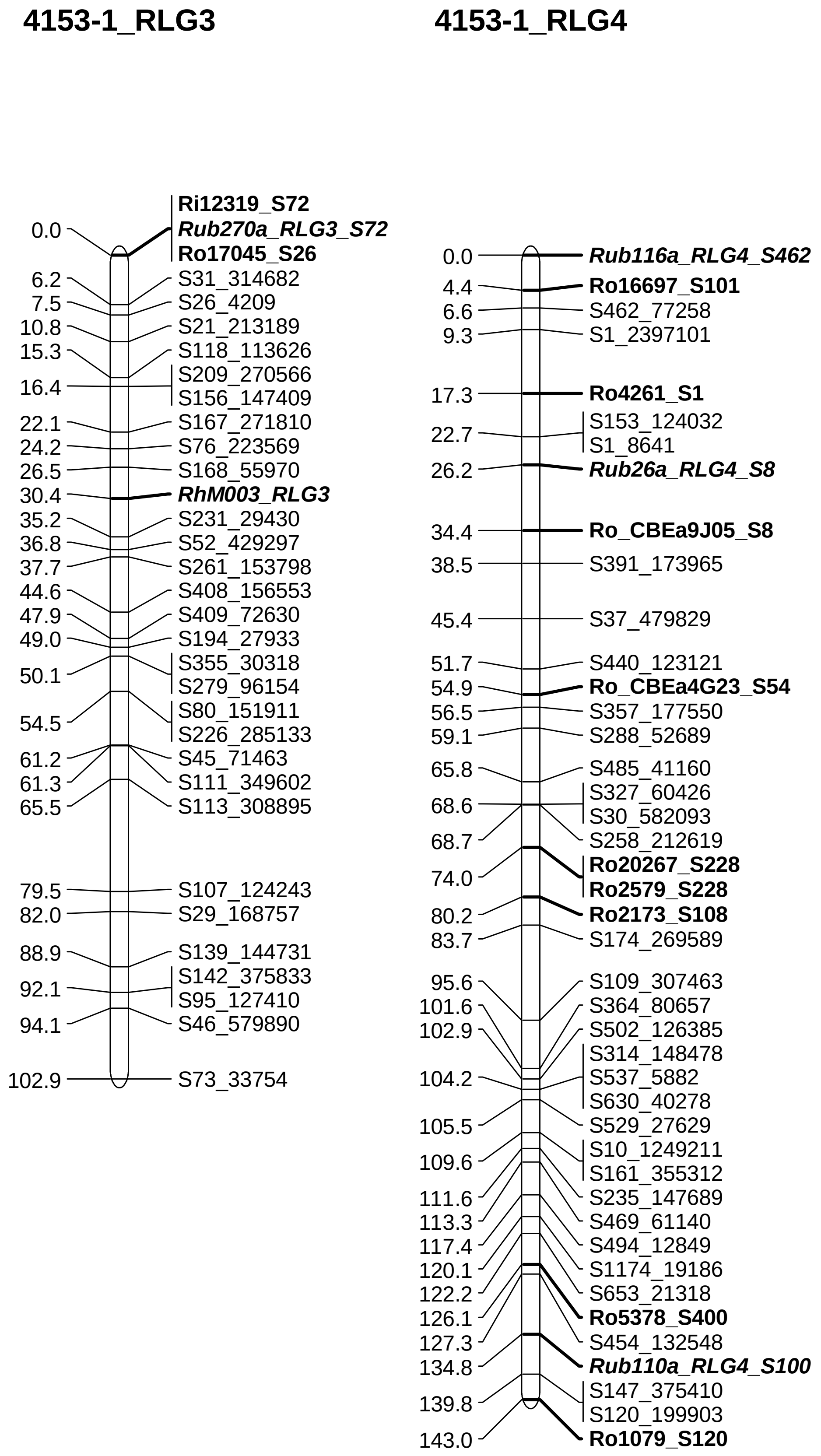

4153-1_RLG3
4153-1_RLG4
Ri12319_S72
0.0 Rub270a_RLG3_S72
Ro17045_S26
6.2 S31_314682
7.5 S26_4209
10.8 S21_213189
15.3 S118_113626
S209_270566
16.4 S156_147409
22.1 S167_271810
24.2 S76_223569
26.5 S168_55970
30.4 RhM003_RLG3
35.2 S231_29430
36.8 S52_429297
37.7 S261_153798
44.6 S408_156553
47.9 S409_72630
49.0 S194_27933
S355_30318
50.1 S279_96154
S80_151911
54.5 S226_285133
61.2 S45_71463
61.3 S111_349602
65.5 S113_308895
79.5 S107_124243
82.0 S29_168757
88.9 S139_144731
S142_375833
92.1 S95_127410
94.1 S46_579890
102.9 S73_33754
0.0 Rub116a_RLG4_S462
4.4 Ro16697_S101
6.6 S462_77258
9.3 S1_2397101
17.3 Ro4261_S1
S153_124032
22.7 S1_8641
26.2 Rub26a_RLG4_S8
34.4 Ro_CBEa9J05_S8
38.5 S391_173965
45.4 S37_479829
51.7 S440_123121
54.9 Ro_CBEa4G23_S54
56.5 S357_177550
59.1 S288_52689
65.8 S485_41160
S327_60426
68.6 S30_582093
68.7 S258_212619
Ro20267_S228
74.0 Ro2579_S228
80.2 Ro2173_S108
83.7 S174_269589
95.6 S109_307463
101.6 S364_80657
102.9 S502_126385
S314_148478
104.2 S537_5882
S630_40278
105.5 S529_27629
S10_1249211
109.6 S161_355312
111.6 S235_147689
113.3 S469_61140
117.4 S494_12849
120.1 S1174_19186
122.2 S653_21318
126.1 Ro5378_S400
127.3 S454_132548
134.8 Rub110a_RLG4_S100
S147_375410
139.8 S120_199903
143.0 Ro1079_S120

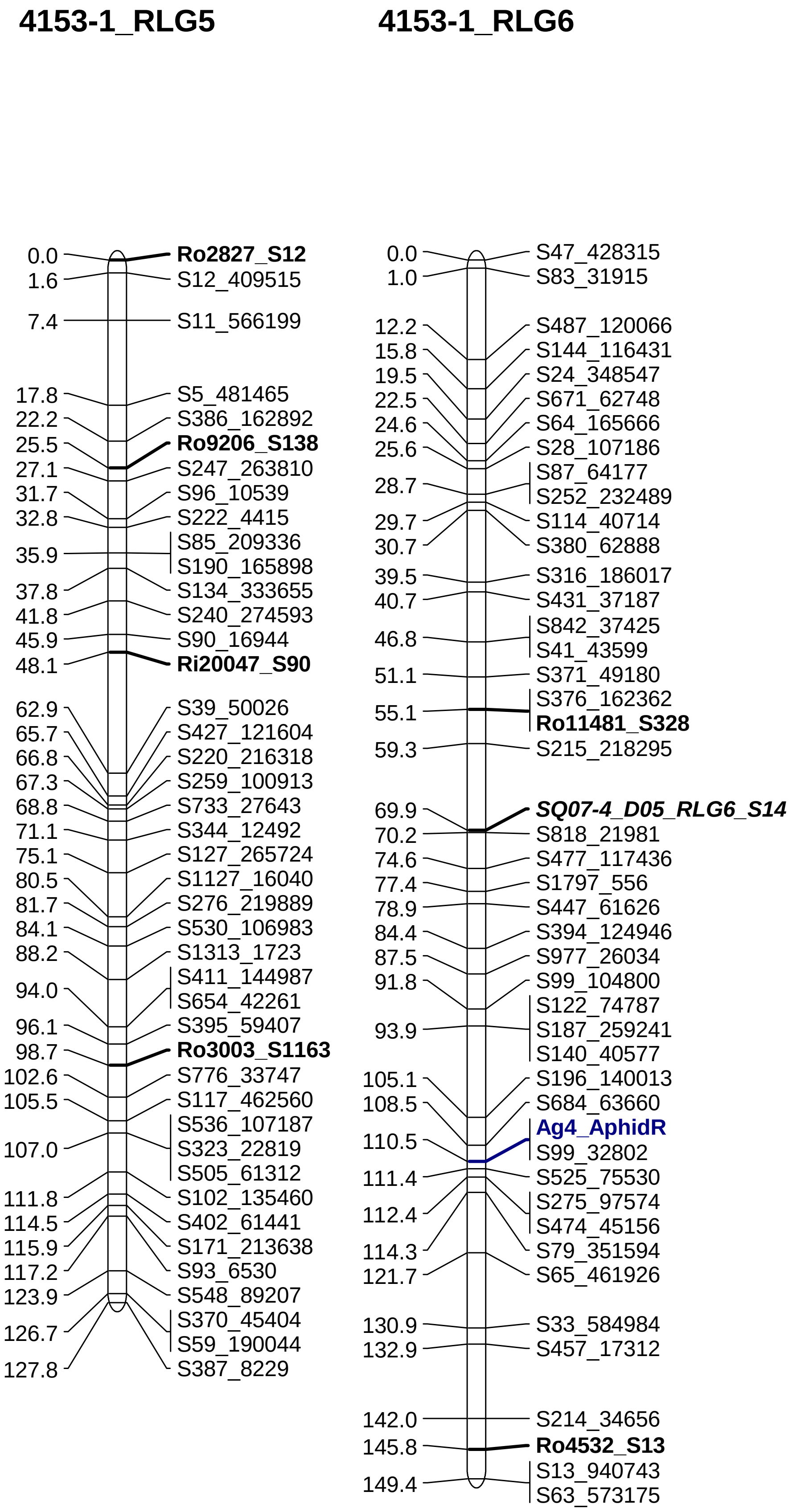
4153-1_RLG5
0.0 Ro2827_S12
1.6 S12_409515
7.4 S11_566199
17.8 S5_481465
22.2 S386_162892
25.5 Ro9206_S138
27.1 S247_263810
31.7 S96_10539
32.8 S222_4415
35.9 S85_209336
S190_165898
37.8 S134_333655
41.8 S240_274593
45.9 S90_16944
48.1 Ri20047_S90
62.9 S39_50026
65.7 S427_121604
66.8 S220_216318
67.3 S259_100913
68.8 S733_27643
71.1 S344_12492
75.1 S127_265724
80.5 S1127_16040
81.7 S276_219889
84.1 S530_106983
88.2 S1313_1723
94.0 S411_144987
S654_42261
96.1 S395_59407
98.7 Ro3003_S1163
102.6 S776_33747
105.5 S117_462560
107.0 S536_107187
S323_22819
S505_61312
111.8 S102_135460
114.5 S402_61441
115.9 S171_213638
117.2 S93_6530
123.9 S548_89207
126.7 S370_45404
S59_190044
127.8 S387_8229
4153-1_RLG6
0.0 S47_428315
1.0 S83_31915
12.2 S487_120066
15.8 S144_116431
19.5 S24_348547
22.5 S671_62748
24.6 S64_165666
25.6 S28_107186
28.7 S87_64177
S252_232489
29.7 S114_40714
30.7 S380_62888
39.5 S316_186017
40.7 S431_37187
46.8 S842_37425
S41_43599
51.1 S371_49180
55.1 S376_162362
Ro11481_S328
59.3 S215_218295
69.9 SQ07-4_D05_RLG6_S14
70.2 S818_21981
74.6 S477_117436
77.4 S1797_556
78.9 S447_61626
84.4 S394_124946
87.5 S977_26034
91.8 S99_104800
93.9 S122_74787
S187_259241
S140_40577
105.1 S196_140013
108.5 S684_63660
110.5 Ag4_AphidR
S99_32802
111.4 S525_75530
112.4 S275_97574
S474_45156
114.3 S79_351594
121.7 S65_461926
130.9 S33_584984
132.9 S457_17312
142.0 S214_34656
145.8 Ro4532_S13
149.4 S13_940743
S63_573175

4153-1_RLG7

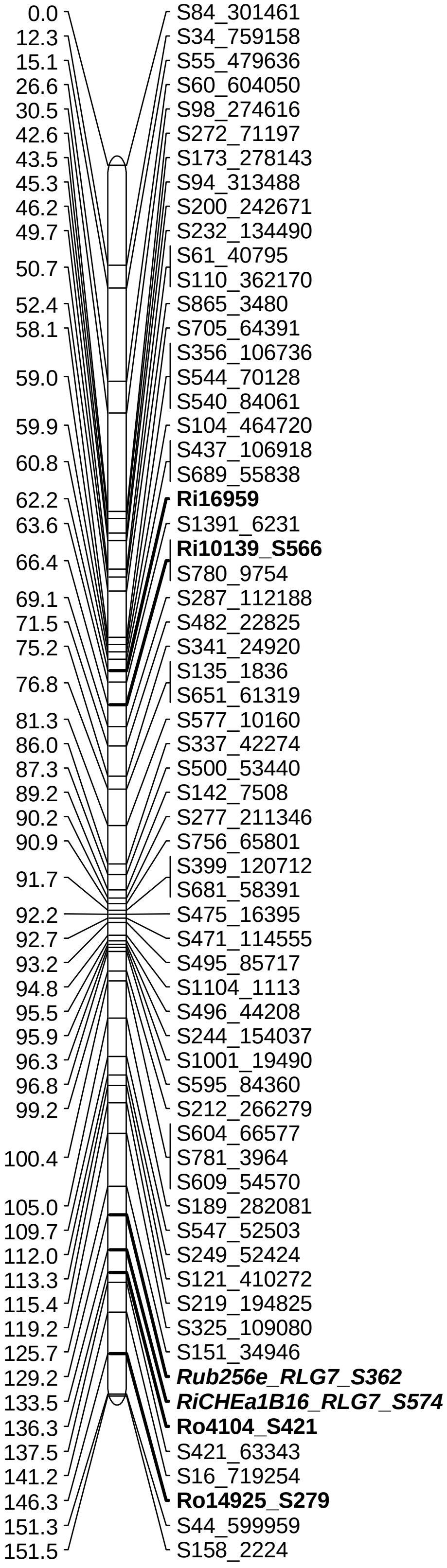
0.0
12.3
15.1
26.6
30.5
42.6
43.5
45.3
46.2
49.7
50.7
52.4
58.1
59.0
59.9
60.8
62.2
63.6
66.4
69.1
71.5
75.2
76.8
81.3
86.0
87.3
89.2
90.2
90.9
91.7
92.2
92.7
93.2
94.8
95.5
95.9
96.3
96.8
99.2
100.4
105.0
109.7
112.0
113.3
115.4
119.2
125.7
129.2
133.5
136.3
137.5
141.2
146.3
151.3
151.5
S84_301461
S34_759158
S55_479636
S60_604050
S98_274616
S272_71197
S173_278143
S94_313488
S200_242671
S232_134490
S61_40795
S110_362170
S865_3480
S705_64391
S356_106736
S544_70128
S540_84061
S104_464720
S437_106918
S689_55838
Ri16959
S1391_6231
Ri10139_S566
S780_9754
S287_112188
S482_22825
S341_24920
S135_1836
S651_61319
S577_10160
S337_42274
S500_53440
S142_7508
S277_211346
S756_65801
S399_120712
S681_58391
S475_16395
S471_114555
S495_85717
S1104_1113
S496_44208
S244_154037
S1001_19490
S595_84360
S212_266279
S604_66577
S781_3964
S609_54570
S189_282081
S547_52503
S249_52424
S121_410272
S219_194825
S325_109080
S151_34946
Rub256e_RLG7_S362
RiCHEa1B16_RLG7_S574
Ro4104_S421
S421_63343
S16_719254
Ro14925_S279
S44_599959
S158_2224
